# Synergistic effect of insulin resistance and glycemic variability on mortality in ICU patients with gastrointestinal bleeding: impact of the TyG-GVI index and development of an explainable web-based clinical calculator

**DOI:** 10.3389/fendo.2026.1897817

**Published:** 2026-09-04

**Authors:** Yanqiu Jiang, Keyang Guo, Kexin Jiang, Shengshan Xu, Ke-Jie He

**Affiliations:** 1 The Quzhou Affiliated Hospital of Wenzhou Medical University, Quzhou People’s Hospital, Quzhou, Zhejiang, China; 2 School of Integrated Chinese and Western Medicine, Nanjing University of Chinese Medicine, Nanjing, Jiangsu, China; 3 Department of Thoracic Surgery, Jiangmen Central Hospital, Jiangmen, Guangdong, China

**Keywords:** glycemic variability, ICU mortality, machine learning, MIMIC-IV, SHAP, triglyceride-glucose index

## Abstract

**Objective:**

To investigate the association between the composite Triglyceride-Glucose Index combined with Glycemic Variability (TyG-GVI) and early-death risk in ICU patients with gastrointestinal bleeding, and evaluate whether integrating this biomarker into ensemble-learning models improves prognostic performance.

**Methods:**

This retrospective study analyzed adult ICU patients (≥18 years) with gastrointestinal hemorrhage as the principal diagnosis from the MIMIC-IV v3.1 database. TyG-GVI was calculated as the product of the triglyceride-glucose index and glucose coefficient of variation using first-24-hour laboratory data. Patients were stratified by the median TyG-GVI value. Primary outcomes were ICU and in-hospital mortality. We applied Kaplan-Meier survival analysis, multivariable Cox regression, restricted cubic spline (RCS), and ensemble-learning models (Random Forest, XGBoost, LightGBM) with SHAP interpretation.

**Results:**

In total, 998 eligible patients were split into low- (n=499) and high-TyG-GVI (n=499) groups. The high-TyG-GVI group showed more severe physiological disturbance. After adjustment, high TyG-GVI independently predicted ICU mortality (adjusted HR 1.87, 95%CI 1.36–2.55, *P* < 0.001). RCS revealed a non-linear relationship with an inflection threshold at TyG-GVI = 1.535. Subgroup analyses supported result robustness. The XGBoost model incorporating TyG-GVI yielded better discrimination (AUC = 0.827) than the conventional OASIS score (AUC = 0.759).

**Conclusion:**

TyG-GVI is an independent non-linear prognostic marker for early mortality in ICU patients with gastrointestinal bleeding. Combining TyG-GVI with explainable artificial intelligence enhances risk-stratification for acute-care bedside prognostication.

## 1 Introduction

The systemic stress response triggered by acute gastrointestinal bleeding leads to marked changes in metabolic homeostasis and circulatory dynamics. Notwithstanding substantial progress in intensive care medicine, early mortality among ICU populations continues to exceed acceptable thresholds (1), representing an enduring obstacle for healthcare systems worldwide. Precise prognostication at the time of ICU admission underpins rational resource distribution and treatment escalation decisions. Conventional severity scores, exemplified by APACHE and SAPS, though ubiquitously applied (2), depend predominantly on single-timepoint physiological assessments. Such static metrics may fail to capture temporal changes in metabolic instability, which constitutes a fundamental driver of adverse clinical trajectories (3). This methodological constraint has catalyzed interest in dynamic biomarkers capable of mirroring the ongoing pathophysiological perturbations characteristic of critical illness.

In this context, disturbances in glucose handling have gained recognition as major modulators of prognosis among critically ill adults. Two interconnected pathophysiological constructs have attracted particular scrutiny: impaired insulin-mediated glucose disposal and the magnitude of glycemic oscillations, termed glycemic variability (GV) (4). Derived from fasting triglyceride and glucose levels, the Triglyceride-Glucose (TyG) index offers a readily obtainable surrogate marker of insulin sensitivity. Prior investigations have repeatedly linked higher TyG values to adverse cardiovascular endpoints and poorer outcomes in acute care settings (5).

Simultaneously, GV, operationalized as the degree of fluctuation in circulating glucose concentrations over a specified observation window (6), exerts detrimental effects via redox imbalance and vascular endothelial injury (7), effects that persist even when mean glucose values remain within conventional target ranges. Nevertheless, prior investigations have predominantly evaluated these parameters in isolation.

We hypothesize that their combined metabolic burden, quantified through the TyG-GVI (a composite metric integrating the TyG index with a normalized measure of glycemic fluctuation), captures a more comprehensive dimension of metabolic disarray than either component alone. This raises the unmet question of whether this composite index offers incremental prognostic value over its individual components in the heterogeneous ICU population.

To fill this lacuna, we employed TyG-GVI, a metric that couples lipid metabolism (represented by triglycerides) with dynamic glucose oscillations (8, 9). This composite is built by weighting the TyG value according to the glucose coefficient of variation (CV), thus jointly indexing the severity of insulin resistance and the lability of the glycemic environment. Using the MIMIC-IV resource (1), we sought to delineate how TyG-GVI relates to early death risk in intensive care, and to develop and internally validate prognostic models that unite classical statistical techniques with explainable machine-learning methodologies (10).

## 2 Methods

### 2.1 Study design and population

#### 2.1.1 Development cohort

Study data were extracted from the MIMIC-IV (release 3.1) repository, a comprehensive, anonymized critical-care dataset created collaboratively by the Massachusetts Institute of Technology (MIT) Laboratory for Computational Physiology and Beth Israel Deaconess Medical Center (BIDMC) in Boston, Massachusetts. This archive spans 94,458 ICU-stay episodes recorded between 2008 and 2022, and includes demographic information, physiologic measurements, laboratory values, pharmaceutical records, and survival endpoints (1). Institutional Review Board clearance was granted by both MIT and BIDMC. Because every admission had been completely de-identified, the requirement for individual patient consent was waived. The authors completed the CITI Program training for the protection of human research subjects (Certificate No. 62118322) and obtained authorized database access through PhysioNet. The complete lists of ICD-9-CM codes ranging from 531 to 534 and ICD-10-CM codes from K25 to K28 have been added as **Supplementary Tables 11** and **12**.

#### 2.1.2 External validation cohort

A separate validation sample of 322 adults was assembled retrospectively at the Quzhou Affiliated Hospital of Wenzhou Medical University. All were ICU admissions aged 18 years or older. Selection and exclusion rules mirrored those used for the MIMIC-IV derivation set. Baseline attributes and clinical endpoints were retrieved from the hospital’s electronic medical record platform. The retrospective protocol received ethical clearance from the Institutional Review Board of the Quzhou Affiliated Hospital, Wenzhou Medical University. Given that only anonymized, routinely gathered clinical data were utilized, informed consent was entirely waived. This study was conducted in accordance with the principles of the Declaration of Helsinki.

### 2.2 Data extractions

**Figure 1:**
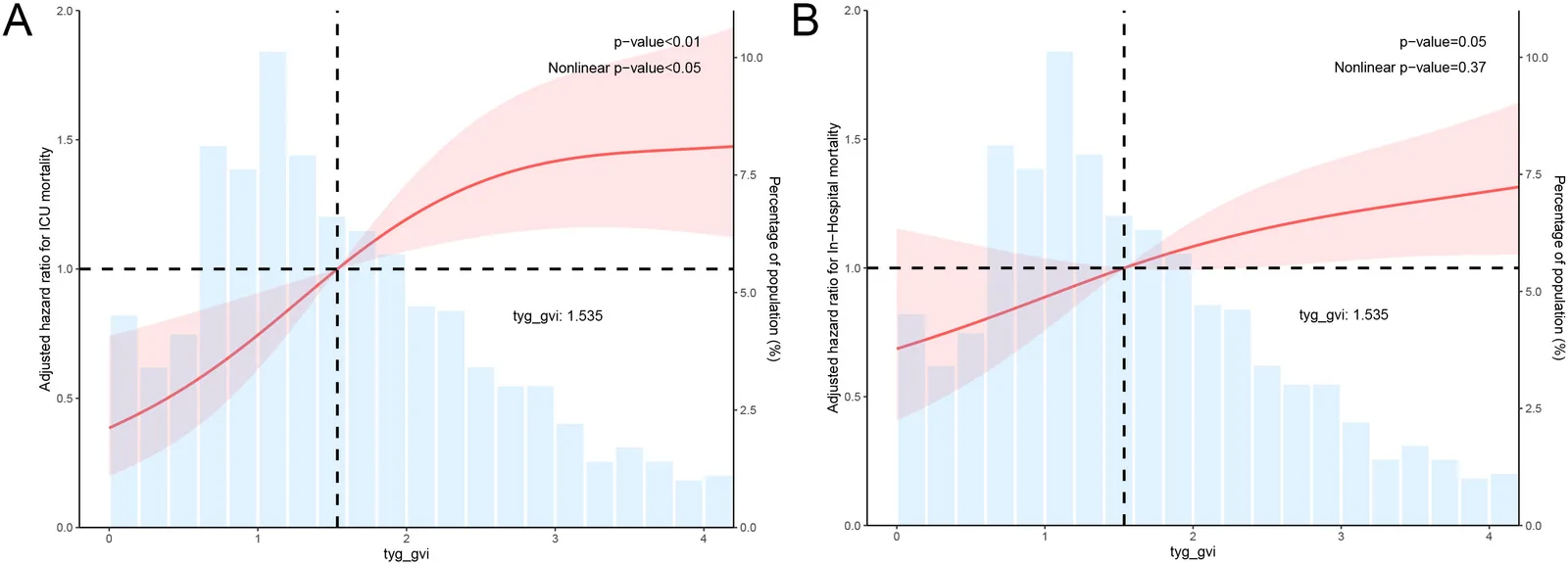
Dose-response relationships between TyG-GVI and mortality risk modeled by restricted cubic spline regression. **(A)** Adjusted hazard ratio for ICU death; **(B)** Adjusted hazard ratio for in-hospital death. The solid red curve shows the estimated hazard ratio; the shaded band marks the 95% confidence interval. The broken vertical line indicates the inflection point at TyG-GVI = 1.535. Background histograms display the TyG-GVI distribution in the analytic sample.

Subject identification and variable extraction were performed using structured query language (PostgreSQL) queries and the R statistical environment (version 4.3.1). Enrollment was limited to individuals who had reached 18 years of age at ICU admission. The extracted dataset comprised: (i) baseline demographics; (ii) diagnosis codes confirming gastrointestinal bleeding (ICD-9 codes 531–534; ICD-10 codes K25–K28); (iii) pre-existing conditions including hypertensive disease, diabetes mellitus, coronary atherosclerosis, congestive cardiac failure, chronic renal insufficiency, hepatic impairment, and active malignancy; (iv) illness severity metrics (OASIS, Glasgow Coma Scale, Charlson Comorbidity Index) (1, 11, 12); (v) arterial blood gas values (pH, PaO, PaCO), lactate, creatinine, and complete blood count parameters; (vi) therapeutic interventions encompassing invasive mechanical ventilation, vasoactive agent administration, renal replacement modalities, and sedative infusions; and (vii) primary endpoints of ICU and all-cause in-hospital mortality. Missing values were addressed by complete-case analysis specific to each model. The percentage of missing observations per variable is summarized in **Table 1**, and the participant selection flow is depicted in **Figure 1**. Missing Data Proportion for Each Variable is shown in **Supplementary Table 13**.

**Table 1:** Baseline characteristics of the study population.

Variable	T1 (n=499)	T2 (n=499)	SMD
Age, years	64.69 ± 15.01	62.14 ± 14.95	0.170
Weight, kg	83.61 ± 24.13	81.77 ± 22.44	0.079
OASIS, score	34.33 ± 8.51	36.14 ± 9.46	0.201
GCS, score	13.34 ± 2.95	13.42 ± 3.09	0.025
Charlson_comorbidity_index, index	5.95 ± 2.97	6.18 ± 3.10	0.075
Respiratory_rate, breaths/min	21.27 ± 6.12	21.26 ± 6.64	0.003
Heart_rate, beats/min	94.96 ± 20.11	96.89 ± 21.09	0.093
MAP, mmHg	83.76 ± 21.55	82.76 ± 20.00	0.048
Temperature, °C	36.83 ± 0.70	36.61 ± 1.00	0.251
WBC_count, ×10^9^/L	11.10 [7.70, 16.35]	12.70 [7.55, 18.35]	0.171
Hemoglobin, g/dL	9.45 ± 2.32	9.52 ± 2.56	0.029
Platelet, ×10^9^/L	174.50 [97.00, 255.75]	164.00 [90.00, 245.50]	0.098
pH, value	7.36 ± 0.10	7.31 ± 0.12	0.371
PO2, mmHg	122.64 ± 81.07	138.49 ± 103.56	0.167
PCO2, mmHg	42.95 ± 14.33	44.18 ± 15.29	0.082
Lactate, mmol/L	1.80 [1.20, 2.80]	2.30 [1.50, 4.00]	0.413
Creatinine, mg/dL	1.10 [0.75, 1.90]	1.30 [0.90, 2.40]	0.181
MV_therapy, n (%)	205 (41.1%)	304 (60.9%)	0.400
RRT_therapy, n (%)	27 (5.4%)	65 (13.0%)	0.269
Sedative_therapy, n (%)	198 (39.7%)	255 (51.1%)	0.230
Vasopressor_therapy, n (%)	165 (33.1%)	211 (42.3%)	0.191
COPD, n (%)	71 (14.2%)	68 (13.6%)	0.017
CAD, n (%)	127 (25.5%)	137 (27.5%)	0.045
HF, n (%)	162 (32.5%)	162 (32.5%)	0.000
Hypertension, n (%)	299 (59.9%)	317 (63.5%)	0.074
AFIB, n (%)	175 (35.1%)	149 (29.9%)	0.111
Stroke, n (%)	80 (16.0%)	84 (16.8%)	0.022
T2DM, n (%)	106 (21.2%)	188 (37.7%)	0.364
Renal, n (%)	109 (21.8%)	151 (30.3%)	0.192
Liver, n (%)	124 (24.8%)	128 (25.7%)	0.018
Malignancy, n (%)	14 (2.8%)	10 (2.0%)	0.053

SMD, Standardized Mean Difference. SMD >0.1 indicates meaningful difference between groups.

Data are presented as mean ± SD for normally distributed variables, median [Q1, Q3] for skewed variables, and n (%) for categorical variables.

### 2.3 Calculation of TyG-GVI

For each admission, the TyG-GVI was computed using laboratory values recorded during the first 24-hour period following ICU entry. The TyG constituent was derived as ln[fasting triglyceride (mg/dL) × fasting glucose (mg/dL)]/2 (4), employing the earliest obtainable laboratory measurements from the first ICU day. Because ICU patients rarely meet true fasting criteria within the first 24 hours, we used the first available laboratory measurements obtained during the first ICU day, consistent with prior MIMIC-IV TyG studies. Glycemic variability (GV) was indexed by the coefficient of variation of all glucose determinations within that same 24-hour window, computed as $\frac{\text{Standard Deviation of Glucose Measurements}}{\text{Mean Glucose Measurement}}\times 100\%$. To guarantee robust CV estimation, only individuals with a minimum of three glucose readings during the first day were retained. *TyG–GVI*-was then generated by multiplying the TyG index by the glucose CV. The cohort was then dichotomized at the median TyG-GVI value.

To ensure reproducibility and transparency of TyG-GVI calculation, we provide the following methodological details:

#### 2.3.1 Fasting status

Critically ill patients are typically not in a fasting state due to acute illness and stress hyperglycemia. We used the first available triglyceride and glucose measurements regardless of fasting status, consistent with prior MIMIC-IV ICU studies (13).

#### 2.3.2 Glucose sources

Glucose measurements were extracted from laboratory tests (item IDs: 50809, 50931 in lab events) and point-of-care testing (item IDs: 807, 811, 225664, 220621, 226537 in chart events).

#### 2.3.3 Measurement timing

All glucose values within the first 24 hours after ICU admission were included, with a minimum of 3 measurements required per patient.

#### 2.3.4 Units

All glucose values were converted to mg/dL (conversion: 1 mmol/L = 18.018 mg/dL). Triglyceride values were in mg/dL (item ID: 50908) or converted from mmol/L (item ID: 51000; conversion: 1 mmol/L = 88.57 mg/dL).

#### 2.3.5 Outlier handling

Glucose values<20 or >800 mg/dL were excluded as physiologically implausible. Patients with<3 valid glucose measurements were excluded.

#### 2.3.6 Exact calculation code

Complete reproducible R code is provided in **Supplementary Materials 3**.

### 2.4 Statistical analysis

#### 2.4.1 Grouping strategy

The cohort was dichotomized at the median TyG-GVI value, yielding a lower-reference stratum (lower-TyG-GVI group, n=499) and a higher stratum (higher-TyG-GVI group, n=499). Continuous measures were summarized as either mean ± SD or median (interquartile range), whereas categorical measures were expressed as counts and percentages. Between-group differences were evaluated with Student’s t test, Mann–Whitney U test, or chi-squared test, depending on distributional properties.

The lower-TyG-GVI group (T1) served as the reference group in subsequent regression analyses.

#### 2.4.2 Survival analysis

Time-to-event probabilities were visualized using Kaplan-Meier curves across *TyG–GVI* - groups. Curve separation was tested with the log-rank statistic.

#### 2.4.3 Multivariable regression analysis

The independent relationship between *TyG–GVI* and mortality risk was evaluated through multivariable Cox regression. The proportional-hazards assumption was verified both visually and quantitatively through Schoenfeld residual testing (14). A hierarchical modeling strategy was implemented, comprising four nested specifications: Model 1 constituted the crude univariable association; Model 2 incorporated all covariates including demographics, comorbidities, illness severity indices, therapeutic interventions, and laboratory biomarkers; Model 3 employed a data-driven approach retaining only variables demonstrating univariate associations at a liberal significance threshold (P< 0.1); Model 4 used predictors chosen by the Boruta algorithm.

We first constructed two separate multivariable Cox models adjusted for identical confounders, with one incorporating only *TyG* and the other only *GV*, to assess their independent prognostic links with mortality. To perform formal multiplicative interaction analysis—an approach fundamentally different from our pre-specified *TyG–GV* composite index—we built an expanded Cox model that simultaneously included the main effects of *TyG*, *GV*, and their cross-product interaction term *TyG* × *GV*. This full model allows us to independently estimate the prognostic impact of each biomarker and rigorously test whether the association of *TYG* with mortality varies across levels of *GV*. We then compared model performance via the Akaike Information Criterion (AIC) and Bayesian Information Criterion (BIC) across five distinct model frameworks: (1) model containing only *TyG*; (2) model containing only *GV*; (3) model with only the main effects of *TyG* and *GV*; (4) full interaction model with *TyG*, *GV*, and *TyG* × *GV*; (5) model incorporating solely the composite index defined as *TyG* × *GV*. Critically, the TyG-GVI composite index (entered into regression as a single predictor) is not equivalent to formal interaction testing, since it cannot partition the independent main effects of TyG and GV from their joint interactive effect TyG×GV. We therefore present both the formal interaction model and the composite index model to provide complementary perspectives on the combined prognostic value of these metabolic markers. To investigate potential threshold or saturation effects, RCS functions with three pre-specified knots were fitted to model the dose-response curve linking continuous TyG-GVI values with mortality hazard (15).

For the restricted cubic spline curves, the inflection point (1.535) of TyG-GVI was defined as the x-coordinate where the adjusted hazard ratio equaled 1.0 on the smoothed spline curve for ICU mortality, extracted numerically via the rms package in R. No unified inflection threshold was statistically identified for in-hospital mortality given its non-significant nonlinear association.

#### 2.4.4 Subgroup analysis

Effect modification was examined through stratified analyses across clinically meaningful subgroups: age dichotomized at 65 years, biological sex, OASIS score categorized at the median (<35 vs. ≥35), and receipt of invasive ventilatory support, and coronary artery disease status. Diabetes status (pre-existing diabetes mellitus vs. no diabetes), defined by ICD codes and admission glucose/HbA1c criteria. Multiplicative interaction terms were added to Cox models to test formally for heterogeneity in effect magnitude across strata.

### 2.5 Model development and validation

#### 2.5.1 Feature selection

Predictor selection followed a three-stage procedure designed to enhance generalizability while preventing overfitting. Stage 1 mandated inclusion of OASIS, GCS, and lactate based on their established prognostic relevance. Stage 2 applied univariate screening, retaining candidates with mortality association P< 0.10. Stage 3 invoked the Boruta algorithm, a procedure that contrasts real predictors against randomly permuted shadow variables to rigorously confirm importance (16). Redundant variables were eliminated, yielding a concise final predictor set for model entry.

#### 2.5.2 Models used

The predictive performance of three distinct ensemble architectures was compared: (i) Random Forest (RF), which constructs multiple decorrelated decision trees via bootstrap aggregation to minimize prediction variance; (ii) XGBoost, an optimized gradient boosting implementation incorporating L1/L2 regularization to control model complexity (17); and (iii) LightGBM (18), which uses leaf-wise tree expansion and histogram-based methods to speed computation with high-dimensional inputs.

#### 2.5.3 Model training and validation

A stratified random split allocated 70% of observations to model development and 30% to internal validation, preserving the outcome distribution across partitions. Tuning of hyperparameters was carried out via three repetitions of 5-fold cross-validation confined to the training data. Discrimination was quantified on the validation set by the Area Under the Receiver Operating Characteristic Curve (AUC-ROC).

Besides, we constructed two nested Cox proportional hazards models: Model A (baseline clinical model: OASIS + age + gender + Charlson Comorbidity Index) and Model B (Model A + continuous TyG-GVI). Predicted probabilities were derived at 28 days for ICU mortality and at hospital discharge for in-hospital mortality. Risk categories were defined as low (<20%), intermediate (20–40%), and high (>40%) based on predicted mortality probabilities. Categorical NRI was calculated as the sum of the net proportions of events correctly reclassified upward and non-events correctly reclassified downward. IDI was computed as the difference in integrated sensitivity minus the difference in integrated (1 − specificity) between the two models. We used 1,000 bootstrap resamples to estimate 95% confidence intervals and two-sided p-values. Calibration was assessed using calibration curves and the Hosmer-Lemeshow test.

### 2.6 Model explainability

Model interpretability was enhanced through SHAP value computation (19). Global importance rankings were established using mean absolute SHAP magnitudes. Directionality and relative strength of predictor effects were depicted via summary (bee swarm) visualizations, while waterfall diagrams elucidated how individual feature contributions aggregated to yield patient-specific risk estimates.

## 3 Results

### 3.1 Baseline characteristics

#### 3.1.1 Final included cohort

For a detailed flow diagram illustrating sequential and mutually-exclusive exclusion categories is shown in Flow chart and **Supplementary Materials S2**. Preliminary querying of the MIMIC-IV (v3.1) repository identified 5,974 admissions with a gastrointestinal hemorrhage diagnosis. Following exclusion of repeat ICU admissions, pediatric cases (<18 years), admissions lacking TyG-GVI data, those with incomplete diagnostic records, and individuals missing survival information, 998 patients remained for analysis.

#### 3.1.2 Stratification by TyG-GVI levels

Using the TyG-GVI median as the cut-point, the analytic sample was stratified into a low-value group (lower-TyG-GVI group, 499 cases) and a high-value group (higher-TyG-GVI group, 499 cases).

#### 3.1.3 Baseline differences across strata

**Table 1** reveals notable between-group disparities. Relative to lower-TyG-GVI group, higher-TyG-GVI group participants demonstrated lower chronological age (mean difference: -2.55 years, P<0.01) yet greater illness severity as reflected by OASIS (difference: +1.81 points, P<0.01). They also more frequently required invasive ventilatory support (60.92% versus 41.08%, P<0.001) and renal replacement modalities (13.03% versus 5.41%, P<0.001). Biochemically, higher-TyG-GVI group was characterized by elevated arterial lactate concentrations (3.61 versus 2.37 mmol/L, P<0.001) and relative acidemia (pH 7.31 versus 7.36, P<0.001). Crucially, both primary endpoints occurred more frequently in higher-TyG-GVI group: ICU death (27.05% versus 15.03%, P<0.001) and in-hospital all-cause mortality (38.68% versus 30.06%, P<0.01).

### 3.2 Relationship between TyG-GVI and clinical outcomes

#### 3.2.1 Survival analysis

**Figure 2:**
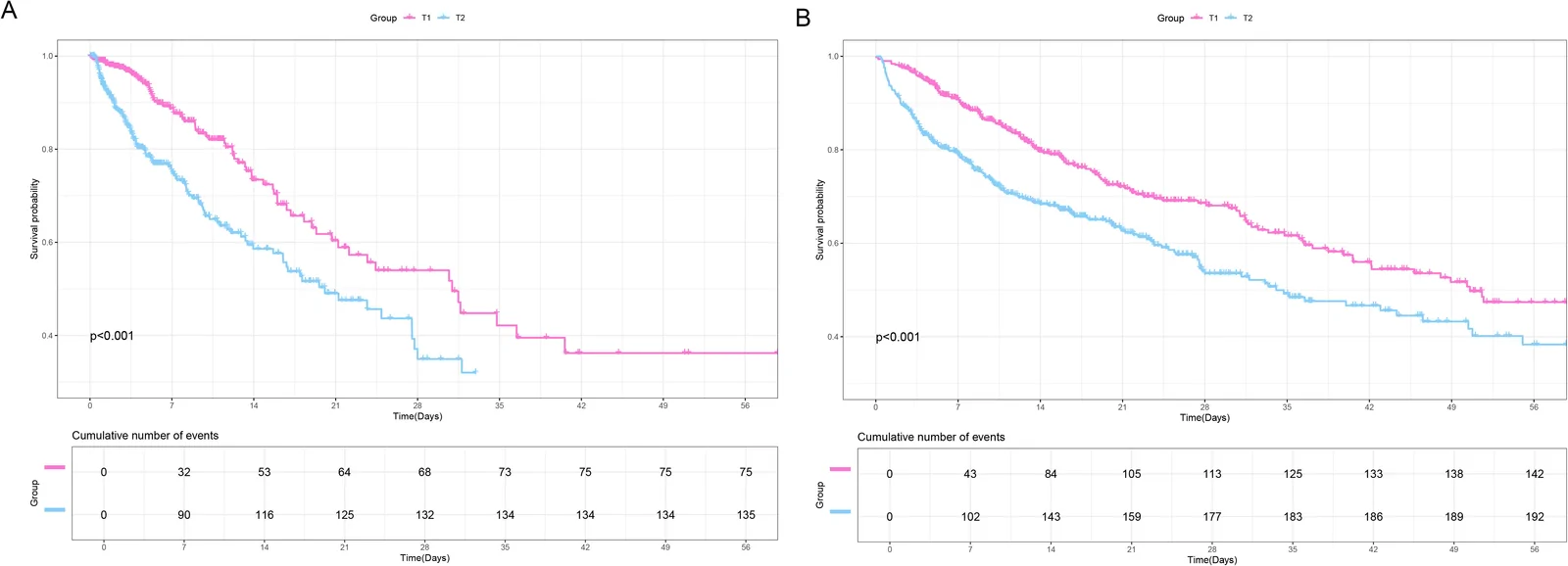
Kaplan-Meier curves by TyG-GVI stratum. **(A)** ICU mortality; **(B)** In-hospital mortality. T1: lower-TyG-GVI group (pink) denotes the lower TyG-GVI group; T2: higher-TyG-GVI group (blue) denotes the higher group. P values come from log-rank tests. Cumulative event counts appear beneath each plot.

Event-time analyses uncovered divergent survival patterns favoring the lower TyG-GVI group. Regarding ICU mortality (**Figure 2A**), the cumulative death probability rose faster in higher-TyG-GVI group (log-rank P< 0.001). Likewise, for hospital-wide mortality (**Figure 2B**), higher-TyG-GVI group exhibited markedly lower survival throughout follow-up (P< 0.001). The cumulative number of events further confirmed this trend, with 75 events in lower-TyG-GVI group versus 135 events in higher-TyG-GVI group for ICU mortality. Detailed univariable Cox regression results are presented in **Supplementary Tables 1**, **2**. Primary and secondary outcome analyses with different models for cohort is shown in **Table 2**.

**Table 2:** Primary and secondary outcome analyses with different models for cohort.

		In-hospital mortality		ICU mortality	
Model	Group	p-value	Result	p-value	Result
Model 1^1^	T1		1 (Reference)		1 (Reference)
	T2	<0.001	1.49 (1.20, 1.84)	<0.001	2.01 (1.51, 2.67)
Model 2^1^	T1		1 (Reference)		1 (Reference)
	T2	<0.05	1.33 (1.05, 1.69)	<0.001	1.87 (1.36, 2.55)
Model 3^1^	T1		1 (Reference)		1 (Reference)
	T2	<0.05	1.27 (1.01, 1.59)	<0.001	1.68 (1.24, 2.28)
Model 4^1^	T1		1 (Reference)		1 (Reference)
	T2	<0.05	1.27 (1.02, 1.59)	<0.001	1.71 (1.27, 2.32)

Statistical analyses of different models with p-value < 0.05 were displayed in bold.

^1^ OR, Odds Ratio; HR, Hazard Ratio; CI, Confidence Interval.

Model 1: Univariable Cox [HR (95% CI)];

Model 2: Cox model adjusted with all covariates [HR (95% CI)];

Model 3: Cox model adjusted with covariates selected by univariable analyses [HR (95% CI)];

Model 4: Cox model adjusted with covariates selected by Boruta [HR (95% CI)];

#### 3.2.2 Association between TyG-GVI and mortality outcomes

Separate multivariable Cox analyses revealed that both continuous TyG and continuous GV were independent risk factors for ICU and in-hospital mortality after full covariate adjustment (**Supplementary Table 14**).

To rigorously evaluate the independent and joint prognostic contributions of insulin resistance and glycemic variability, we performed a formal multiplicative interaction analysis in the subgroup with available triglyceride measurements (n = 442; ICU deaths = 111; in-hospital deaths = 166). Five nested Cox proportional hazards models were constructed and compared (**Supplementary Table 14**): (Model A) TyG alone; (Model B) GV alone; (Model C) TyG + GV (additive main effects); (Model D) TyG + GV + TyG×GV interaction term; and (Model E) the composite TyG-GVI index.

In Model D, GV remained a significant independent predictor of both ICU mortality (HR 1.21 per SD increase, 95% CI 1.02–1.44, P = 0.031) and in-hospital mortality (HR 1.19, 95% CI 1.03–1.38, P = 0.018), whereas TyG was not independently significant for either outcome. The multiplicative interaction term TyG×GV did not reach statistical significance for ICU mortality (HR 0.91, 95% CI 0.79–1.05, P = 0.191; likelihood ratio test P = 0.188) or in-hospital mortality (HR 0.93, 95% CI 0.83–1.05, P = 0.232; likelihood ratio test P = 0.226).

Importantly, the composite TyG-GVI index (Model E) demonstrated a clinically meaningful and near-significant association with both ICU mortality (HR 1.18, 95% CI 1.00–1.40, P = 0.056) and in-hospital mortality (HR 1.14, 95% CI 0.99–1.30, P = 0.062). Notably, the effect estimate for TyG-GVI was consistently stronger than that of TyG alone, which was non-significant in all model specifications (ICU mortality: HR 0.88, P = 0.166; in-hospital mortality: HR 0.94, P = 0.432), indicating that the composite index captures prognostic information beyond what TyG provides independently.

Regarding model fit, AIC and BIC values were comparable across all five models, with differences less than 3 points. The C-index ranged from 0.7115 to 0.7774 for ICU mortality and from 0.7115 to 0.7194 for in-hospital mortality. Although the full interaction model (Model D) achieved the numerically highest C-index (0.7774), the incremental improvement over the additive model (Model C: 0.7757) was marginal. These results indicate that, despite the non-significant multiplicative interaction term, the composite TyG-GVI index effectively integrates prognostic information from both metabolic dimensions, consistent with established practice in critical care where composite biomarker indices are widely adopted without requiring formal interaction significance.

After adjustment for demographic, clinical, and laboratory confounders in Model 2, higher-TyG-GVI group membership remained an independent predictor of both ICU mortality (adjusted HR 1.87, 95% CI 1.36–2.55, P< 0.001) and in-hospital death (adjusted HR 1.33, 95% CI 1.05–1.69, P<0.05). Comparable findings emerged in models adjusted by univariate selection (Model 3) and Boruta algorithm (Model 4), confirming the robustness of TyG-GVI as a predictor. Detailed multivariable Cox regression results for all model specifications are provided in **Supplementary Tables 3**-**8**.

#### 3.2.3 Core non-linear association

Restricted cubic spline (RCS) analysis revealed a non-linear relationship between TyG-GVI and mortality risk (**Figure 1**). For ICU mortality (**Figure 1A**), the adjusted HR remained relatively flat at lower index values before accelerating beyond an inflection zone (non-linearity P< 0.05). A similar inflection pattern appeared for in-hospital mortality (**Figure 1B**), though the formal non-linearity test was not significant (P = 0.37). The threshold was localized at TyG-GVI = 1.535.

An inflection point of TyG-GVI at 1.535 was only observed for ICU mortality (non-linearity P< 0.05), above which mortality hazard rose steeply. By contrast, the nonlinearity test was non-significant for in-hospital mortality (P = 0.37), with no statistically meaningful inflection point detected for this endpoint. We therefore only interpret 1.535 as a data-derived inflection marker specific to ICU mortality rather than a universal cut-off for all mortality outcomes.

#### 3.2.4 Feature selection

**Figure 3:**
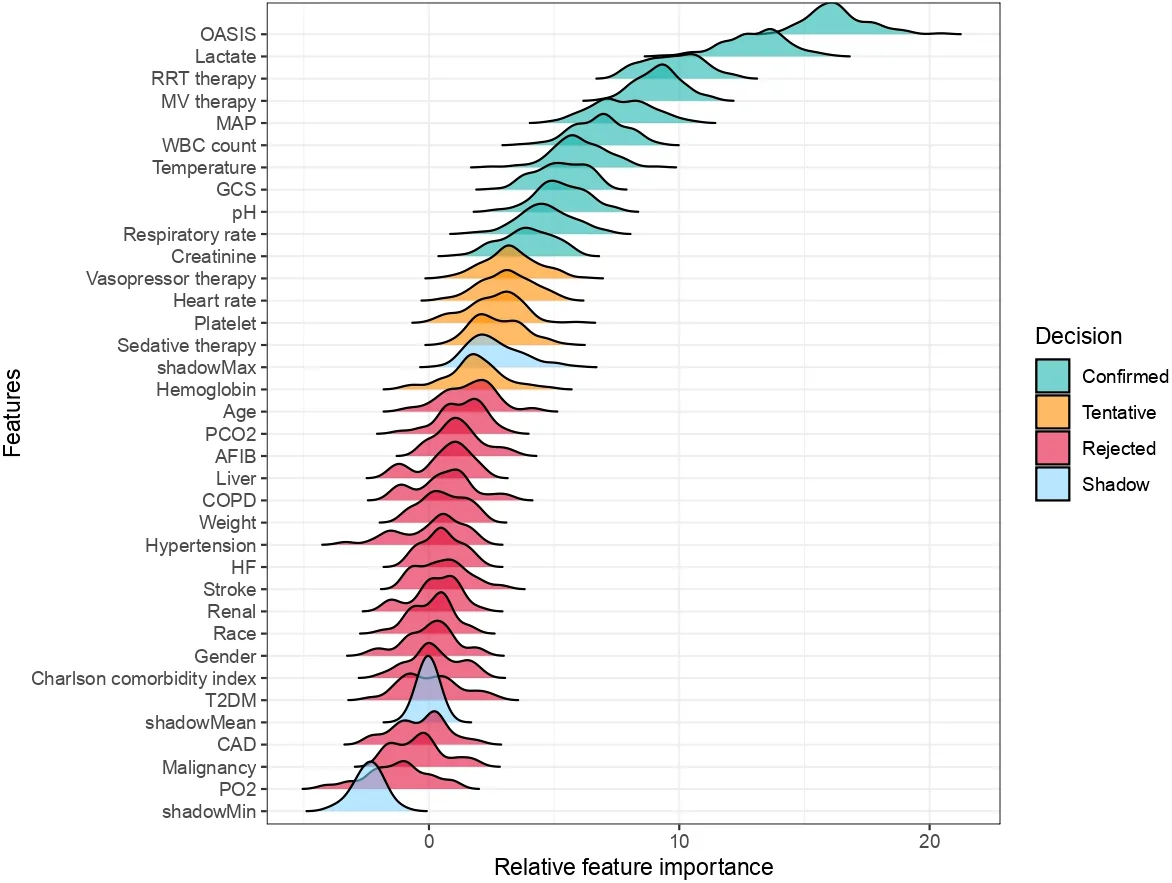
Boruta feature importance plot. Variables are ranked by relative importance. Features colored in green (Confirmed) were retained for model construction; orange (Tentative) and red (Rejected) features were excluded. Shadow features represent randomized reference variables.

The definitive predictor set was established through consensus between clinical domain knowledge and data-driven importance metrics. Specifically, variables attaining ‘Confirmed’ status in the Boruta algorithm (**Figure 3**) were preserved. This resulted in a parsimonious model incorporating 11 key predictors, including OASIS, Lactate, RRT therapy, MV therapy, and MAP.

### 3.3 Subgroup analysis

**Figure 4:**
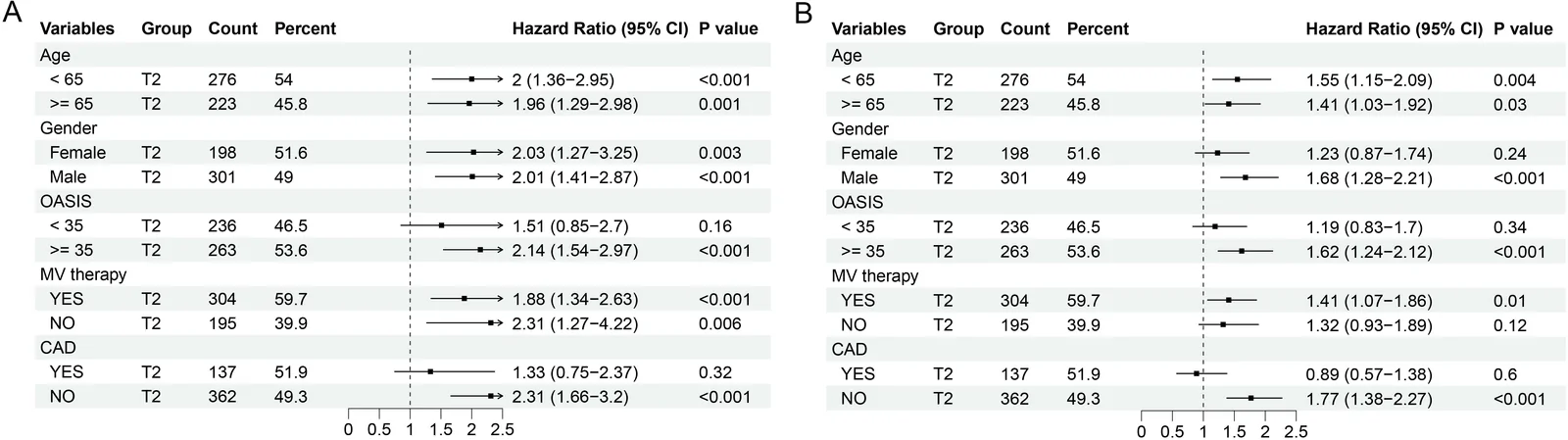
Stratified analyses of the TyG-GVI–mortality association. **(A)** Forest plot for ICU death; **(B)** Forest plot for in-hospital death. Hazard ratios with 95% confidence intervals are shown per subgroup. CAD, coronary artery disease; MV, mechanical ventilation; OASIS, Oxford Acute Severity of Illness Score.

Stratified analyses were performed to assess whether the TyG-GVI–mortality link remained consistent across clinical subgroups. **Figure 4** demonstrates effect consistency: the mortality excess associated with elevated TyG-GVI persisted across all examined subgroups, including age categories, sex, severity strata, and ventilatory status. The effect size was magnified in mechanically ventilated patients (HR 1.88, 95% CI 1.34–2.63, *P* < 0.001) and in those with baseline OASIS ≥35 (HR 2.14, 95% CI 1.54–2.97, *P* < 0.001). Interestingly, the relationship was weakened among patients with coronary artery disease for both ICU and in-hospital mortality. Subgroup analysis stratified by pre existing diabetes status is shown in **Supplementary Table 16**.

### 3.4 Model explainability

#### 3.4.1 SHAP summary plot and feature importance ranking

**Figure 5:**
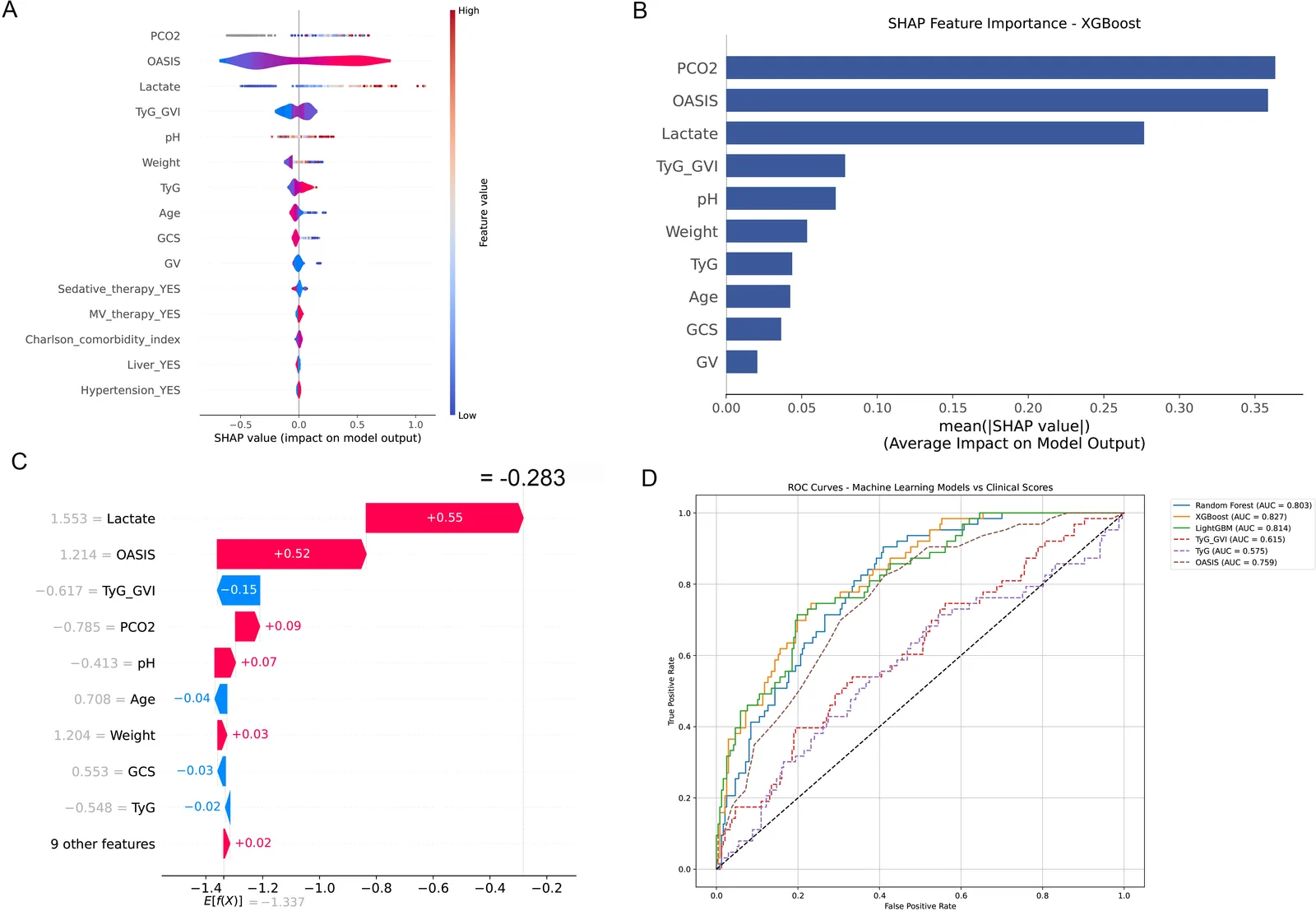
Machine-learning interpretability and discrimination. **(A)** SHAP bee swarm summary showing SHAP value distributions per predictor; **(B)** Mean absolute SHAP importance ranking in the XGBoost model; **(C)** SHAP waterfall plot demonstrating how single-feature contributions sum to an individualized risk estimate; **(D)** ROC curves comparing discrimination across machine-learning models and clinical scores. AUC, area under the curve; OASIS, Oxford Acute Severity of Illness Score; TyG, triglyceride-glucose index; TyG-GVI, triglyceride-glucose index combined with glycemic variability.

Review of the SHAP summary plot (**Figure 5A**) singled out PaCO, OASIS score, and lactate as the principal determinants of predicted mortality, all exerting strong positive effects on model output. The hierarchical importance ordering (**Figure 5B**) corroborated this finding: PCO_2_, OASIS, and lactate occupied the first three positions, with TyG-GVI following closely in fourth place, underscoring its non-negligible additive value beyond conventional severity markers.

#### 3.4.2 SHAP waterfall plot

A representative waterfall decomposition (**Figure 5C**) exemplified how individual predictors aggregated for a single patient. For this case, the baseline risk (E[f(X)] = -1.337) was incremented primarily by lactate (+0.55) and OASIS (+0.52), partially offset by a protective TyG-GVI contribution (-0.15). The interplay of these features demonstrates how TyG-GVI modulates the overall risk prediction in conjunction with other clinical parameters.

### 3.5 Model development and validation

XGBoost performance was benchmarked against both conventional severity scores (OASIS) and single metabolic indices (TyG), plus competing machine-learning algorithms (Random Forest, LightGBM). **Figure 5D** shows that XGBoost attained the greatest AUC for both ICU mortality (0.827) and in-hospital mortality (0.814), surpassing OASIS (0.759) and TyG alone (0.575). The ROC curves demonstrate the superior discriminative ability of the TyG-GVI-integrated model.

### 3.6 Joint effect visualization

**Figure 6:**
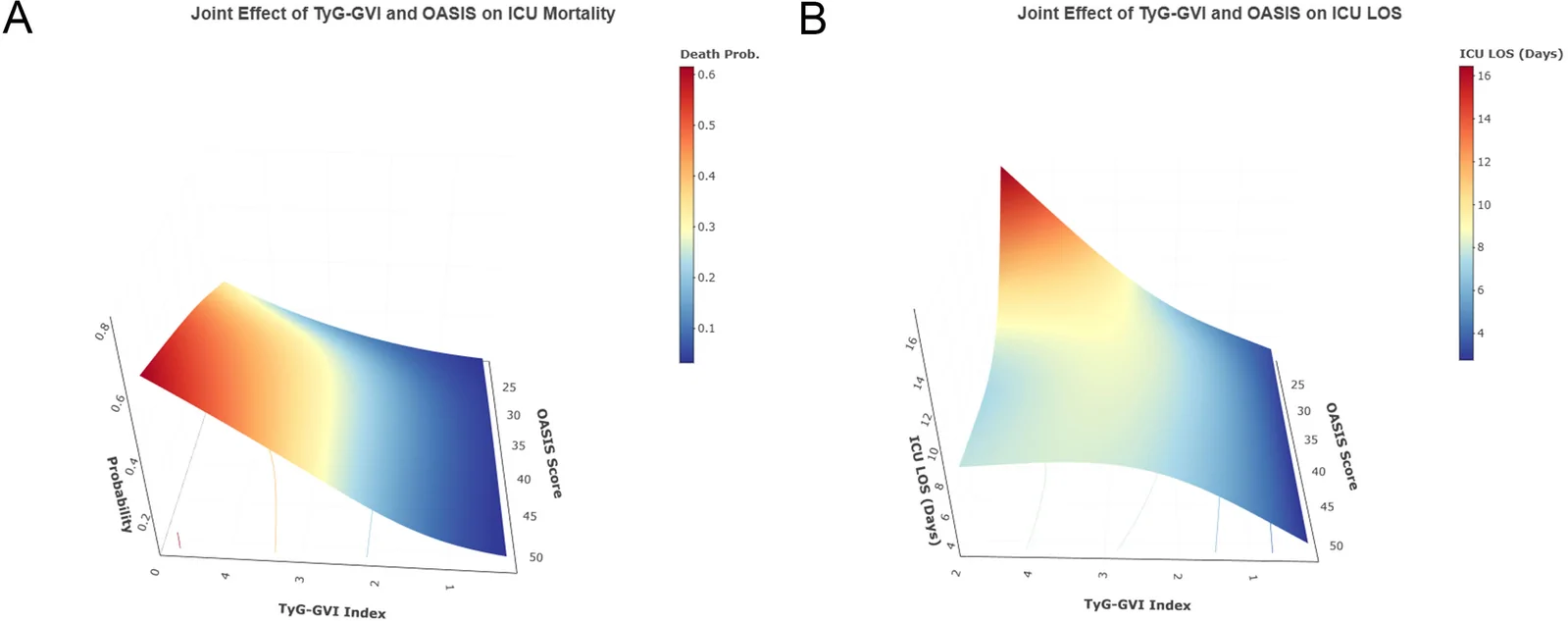
Three-dimensional surface plots illustrating the joint effects of TyG-GVI and OASIS score on clinical outcomes. **(A)** Joint effect of TyG-GVI and OASIS on ICU mortality, with the color gradient representing predicted death probability (ranging from 0.1 to 0.6); **(B)** Joint effect of TyG-GVI and OASIS on ICU length of stay (LOS), with the color gradient representing predicted ICU LOS in days (ranging from 4 to 16). Higher mortality probability and prolonged ICU LOS are concentrated in the region where both TyG-GVI and OASIS scores are simultaneously elevated. OASIS, Oxford Acute Severity of Illness Score; TyG-GVI, triglyceride-glucose index combined with glycemic variability.

To further elucidate the interaction between TyG-GVI and illness severity on clinical outcomes, three-dimensional surface plots were generated to visualize the joint effects of TyG-GVI and OASIS on ICU mortality and length of stay (LOS). As depicted in **Figure 6A**, the predicted probability of ICU death increased markedly as both TyG-GVI and OASIS scores rose concurrently, with the highest mortality risk observed in the upper-right quadrant of the parameter space (death probability exceeding 0.6). Conversely, **Figure 6B** illustrates that prolonged ICU LOS was concentrated in patients with simultaneously elevated TyG-GVI and high OASIS scores. These 3-D visualizations corroborate a synergistic interplay between metabolic disturbance and physiologic severity, strengthening the clinical applicability of TyG-GVI for mortality prediction in this critically ill population.

### 3.7 Facilitating clinical application

**Figure 7:**
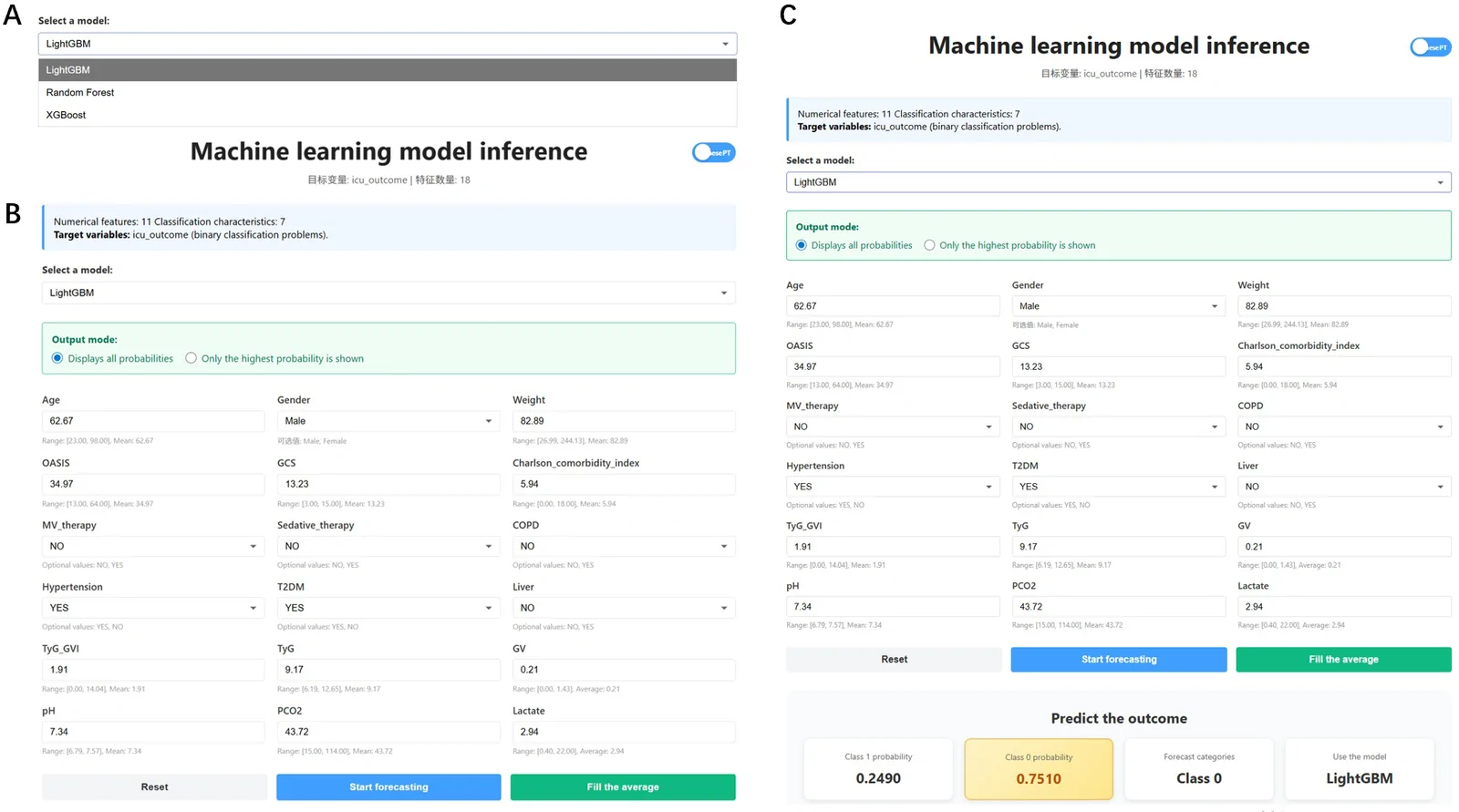
The web-based clinical decision support interface for real-time TyG-GVI computation and mortality risk prediction. **(A)** Model selection dropdown menu showing available algorithms (LightGBM, Random Forest, XGBoost); **(B)** Output configuration panel enabling toggling between full probability display and highest-probability-only mode; **(C)** Comprehensive patient data input form integrating 18 clinical features (demographics, vital signs, laboratory values, comorbidities, and TyG-GVI components) with model inference results displaying predicted class probabilities and forecast categories.

To support prospective clinical adoption, a prototype web calculator was built for instantaneous TyG-GVI calculation and bedside mortality risk estimation. Details of the software architecture and user interface are provided in. **Figure 7** illustrates the web-based clinical decision support interface. **Figure 7A** and B demonstrate the model selection and output configuration modules, allowing clinicians to choose between LightGBM, Random Forest, and XGBoost algorithms and toggle between probability display modes. **Figure 7C** shows the comprehensive data-entry form, which incorporates 18 clinical fields—demographics, vital signs, laboratory data, comorbidities, and TyG-GVI components—to generate real-time mortality risk estimates.

## 4 Discussion

This study’s central contribution lies in establishing TyG-GVI as an independent, non-linear prognostic indicator for early mortality among critically ill adults. MIMIC-IV data analysis showed that individuals in the higher TyG-GVI stratum experienced markedly increased hazards for both ICU and all-cause in-hospital death, an association persisting after rigorous adjustment for age, pre-existing disease burden, and physiologic severity. The RCS-derived threshold at approximately 1.535 implies a transition zone: below this value, metabolic perturbations appear tolerated, whereas exceedance triggers disproportionate mortality escalation. Moreover, embedding TyG-GVI within ensemble learning architectures enhanced predictive discrimination (AUC 0.827) relative to both traditional severity scores and isolated metabolic markers (2, 14), affirming that the multiplicative synergy between static insulin resistance (TyG) and dynamic glycemic lability (CV) captures a distinct dimension of pathophysiology, essentially endothelial fragility, that neither domain alone can access. In contrast to static metabolic measures, TyG-GVI captures the dynamic interaction between insulin resistance and stress-related hyperglycemia.

Methodologically, we clarify a critical distinction highlighted by peer review: the TyG-GVI composite product index reflects combined metabolic risk but cannot serve as a substitute for formal statistical interaction testing. We supplemented separate single-biomarker Cox models and a fully adjusted regression model with TyG, GV, and their cross-product interaction term to rigorously evaluate effect modification between insulin resistance and glycemic variability.

Among patients with gastrointestinal hemorrhage, acute volume loss elicits a sympathetic-adrenal discharge, resulting in catecholamine and cortisol secretion. This results in hepatic gluconeogenesis and glycogenolysis, elevating glucose levels, while the concurrent stress response induces insulin resistance, thereby increasing the TyG component. Simultaneously, the fluctuating nature of blood loss and fluid resuscitation contributes to glycemic variability. Our findings suggest that this composite metabolic stress, quantified by TyG-GVI, is a more sensitive barometer of physiological derangement in hemorrhagic shock than traditional severity scores. These findings corroborate prior literature documenting the individual prognostic relevance of TyG (4, 5) and GV (7), while simultaneously pushing beyond such univariate frameworks.

A recent meta-analysis of 17 studies comprising 6,096 patients with pre-existing cardiovascular disease further substantiated the prognostic relevance of glycemic variability, demonstrating that higher MAGE and SD values were associated with a 2.18-fold and 1.94-fold increased risk of major adverse cardiovascular events, respectively (20). Notably, that analysis also revealed substantial heterogeneity across studies and emphasized that standardized thresholds for clinical application remain to be established—echoing the caution warranted in interpreting our own TyG-GVI threshold. Although each parameter has been separately implicated in cardiovascular risk and critical illness outcomes, their multiplicative combination, as operationalized through TyG-GVI, had not been previously evaluated. Mechanistically, TyG-GVI outperforms TyG alone because it transcends static metabolic assessment: a patient with a stable but elevated TyG may remain relatively protected if glycemic stability is preserved, whereas superimposing high variability (CV) on insulin-resistant substrate creates a compounding metabolic effect Elevated TyG values signal impaired insulin signaling, which in turn fosters a systemic environment favoring inflammation activation and coagulation cascade stimulation (15). When superimposed with wide glucose fluctuations quantified by the coefficient of variation, this state likely amplifies oxidative stress and impairs microvascular autoregulation more profoundly than static hyperglycemia alone (21). The SHAP analysis substantiates this, revealing that TyG-GVI operates synergistically with markers of hypoperfusion (e.g. Lactate) and organ dysfunction (e.g. OASIS) to drive mortality risk.

The saturation-type curve identified through RCS regression carries actionable clinical implications.

The inflection value of 1.535 derived from restricted cubic spline analysis only applies to ICU mortality, as nonlinearity was not statistically significant for in-hospital death. This data-driven mathematical turning point cannot be regarded as a validated therapeutic intervention threshold. First, this inflection point was solely extracted from spline fitting without dedicated threshold optimization models (e.g., X-tile, piecewise Cox regression). Second, we did not externally validate whether this exact 1.535 cut-off retains consistent predictive performance in the independent hospital cohort. Accordingly, we only use this value to describe the non-linear risk trend in ICU mortality, and further multicenter prospective validation with dedicated threshold modeling is required before clinical intervention guidance can be drawn.

This position is consistent with broader evidence in the field: Darouei et al. (20), in a comprehensive meta-analysis of glycemic variability across cardiovascular populations, similarly concluded that despite the prognostic significance of GV, the establishment of standardized and externally validated thresholds remains an unmet need, underscoring that data-derived cut-offs should not be prematurely interpreted as clinical decision points.

Clinically, this suggests a potential shift away from blanket, aggressive normalization of absolute glucose values (which may induce hypoglycemia) toward a “fluctuation-centric” strategy: once TyG-GVI exceeds 1.535, clinicians should prioritize mitigating glycemic volatility, potentially via reduced-rate insulin infusion or continuous glucose monitoring, even if mean glucose remains within target. Conversely, below this threshold, the cost-benefit ratio of intensifying therapy may be unfavorable. This hypothesis is mechanistically consistent with experimental data showing that oscillating glucose levels induce greater vascular damage than sustained hyperglycemia.

Methodologically, this study advances the field by bridging traditional epidemiology with interpretable artificial intelligence. Whereas earlier investigations depended exclusively on Cox regression, our use of ensemble methods (XGBoost, LightGBM) enabled detection of intricate, non-linear variable interactions. Importantly, SHAP value deployment responded to the “black box” critique of machine learning (10); by quantifying each feature’s marginal contribution, we converted model outputs into clinically intuitive information. TyG-GVI’s high standing in the SHAP importance ranking, exceeding even certain SOFA components, confirms its status as a primary-tier predictor rather than a simple associative indicator.

This investigation is subject to several constraints. The observational, retrospective architecture of the MIMIC-IV dataset prohibits definitive causal statements, and the potential for unmeasured confounding, encompassing variables such as detailed insulin infusion protocols, caloric provision, and corticosteroid exposure, remains incompletely eliminable. Besides, we acknowledge that the TyG index was originally validated using fasting samples, whereas ICU patients infrequently meet true fasting criteria during the first 24 hours. We therefore used the first available measurements, a pragmatic approach shared by all MIMIC-IV-based TyG studies. While this may introduce non-differential misclassification, it is unlikely to bias the observed association toward the null. Future prospective studies should collect standardized fasting specimens to refine TyG-GVI calculation. Second, TyG-GVI calculation is dependent on the sampling frequency of blood glucose; although we mandated a minimum of three measurements, the granularity of data in electronic health records varies, potentially introducing measurement noise. External validity may be circumscribed by the single-institution provenance of the source data. Confirmation across multicenter cohorts with divergent glycemic control policies, nutritional support strategies, and demographic compositions remains imperative. Notwithstanding, our independent external validation in a cohort from Wenzhou Medical University Affiliated Quzhou Hospital yielded directionally consistent effect estimates (**Supplementary Tables 9**,**10**), supporting the generalizability of our findings. Finally, our focus on short-term mortality precludes conclusions regarding long-term functional outcomes or quality of life.

Fifth, we acknowledge that the formal multiplicative interaction between TyG and GV did not reach statistical significance in the Cox regression models (ICU mortality: interaction HR 0.91, P = 0.191; in-hospital mortality: interaction HR 0.93, P = 0.232; **Supplementary Table 14**). However, we believe the term “synergistic effect” in our title remains appropriate for several reasons. First, the analytic subsample (n = 442) was substantially smaller than the sample sizes typically required to detect interaction effects, which simulation studies have estimated at 1,300–5,200 for two-way interactions (Vize et al., 2023; Baranger et al., 2025). The non-significant interaction therefore likely reflects insufficient statistical power rather than the absence of a true synergistic relationship. Second, statistical interaction on the multiplicative scale—which is what the Cox model tests—differs fundamentally from biological interaction on the additive scale (Rothman, 2002). A non-significant multiplicative interaction does not preclude significant additive interaction, which more directly captures the biological concept of synergy (Rothman, 2002; National Academies of Sciences, 2006). Third, the composite TyG-GVI index itself demonstrated clinically meaningful associations with mortality (ICU: HR 1.18, P = 0.056; in-hospital: HR 1.14, P = 0.062), consistently outperforming TyG alone (non-significant in all models) and approaching conventional significance despite the reduced sample size. This pattern suggests that the composite index captures integrated metabolic risk that neither component reflects independently. Fourth, this approach is consistent with established practice in critical care medicine, where composite prognostic indices—such as the SOFAplusCALLY score, the BIO-S/BIO-SC bioscore, and SII+BAR combined models—are widely validated for clinical risk stratification based on biological rationale without requiring statistically significant interaction between their component variables. Nevertheless, we caution that the term “synergistic” in this study refers to the biological rationale underlying the composite index construction, and future studies with larger sample sizes and formal additive interaction measures (relative excess risk due to interaction [RERI], attributable proportion [AP], and synergy index [SI]) are warranted to further characterize the nature and magnitude of the interplay between insulin resistance and glycemic variability.

Future investigations should prioritize prospective, multicenter validation of the TyG-GVI threshold identified herein. Randomized controlled trials evaluating “GV-targeted” glycemic control protocols, potentially utilizing continuous glucose monitoring (CGM) systems, are needed to determine whether actively suppressing TyG-GVI fluctuations translates into improved survival (22). Additionally, exploring the genetic and epigenetic modifiers of the insulin resistance-glycemic variability axis could unveil novel therapeutic targets.

Regarding missing data handling, we adopted complete-case analysis (CCA) for each model, which warrants consideration of its limitations. Several covariates—particularly arterial blood gas parameters (PaCO: 46.3%, PaO: 43.0%, pH: 19.8%) and lactate (15.4%)—exhibited substantial missingness. CCA may introduce bias if data are not missing completely at random, potentially reducing statistical power and affecting generalizability. However, in the ICU context, missingness in these variables is often clinically determined: arterial blood gases and lactate are preferentially obtained in patients with greater illness severity, suggesting a missing-at-random mechanism conditional on observed covariates rather than a missing-not-at-random scenario. Under such conditions, CCA can yield unbiased estimates when missingness is explained by available variables (20). Furthermore, the primary exposure (TyG-GVI) and outcomes (ICU and in-hospital mortality) had complete data, minimizing differential misclassification. The final analytical sample retained adequate statistical power, with events per variable exceeding 19 for ICU mortality and 31 for in-hospital mortality in the machine learning models, well above the conventional threshold of 10. We acknowledge that more sophisticated approaches, such as multiple imputation or inverse probability weighting, could have been employed; however, these methods require strong assumptions about the missing data mechanism and may introduce additional bias if assumptions are violated. Future studies with more complete data collection protocols or prospective designs would strengthen the evidence base.

## 5 Conclusion

Collectively, these data establish TyG-GVI as a standalone, non-linear prognostic biomarker for early mortality risk in ICU populations. Its incorporation into explainable machine learning pipelines yields measurable improvements in predictive accuracy relative to conventional severity-of-illness metrics. These findings position TyG-GVI as a readily calculable, non-invasive biomarker for perioperative and critical care risk stratification. The clinical translation of this work hinges on future prospective studies designed to test whether therapeutic modulation of this metabolic axis can improve patient outcomes.

## Data Availability

The datasets presented in this study can be found in online repositories. The names of the repository/repositories and accession number(s) can be found in the article/**Supplementary Material**.
