# Supplementary material for: Synergistic effect of insulin resistance and glycemic variability on mortality in ICU patients with gastrointestinal bleeding: impact of the TyG-GVI index and development of an explainable web-based clinical calculator
Source: Front Endocrinol (Lausanne). 2026 Sep 4;17:1897817. doi: 10.3389/fendo.2026.1897817 (PMC13585509; doi:10.3389/fendo.2026.1897817)
Supplement: Supplementary file 1 [file SupplementaryFile1.docx]

Supplementary Table 1. Time Dependent univariable Cox model for ICU mortality of mimic4_v31 cohort

| **Characteristic** | **HR**^1^ | **95% CI**^1^ | **p-value** |
| --- | --- | --- | --- |
| Group |  |  |  |
| T1 | 1.00 | Reference |  |
| T2 | 2.01 | 1.51, 2.67 | <0.001 |
| ^1^HR = Hazard Ratio, CI = Confidence Interval | | | |

Supplementary Table 2. Time Dependent univariable Cox model for In-hospital mortality of mimic4_v31 cohort

| **Characteristic** | **HR**^1^ | **95% CI**^1^ | **p-value** |
| --- | --- | --- | --- |
| Group |  |  |  |
| T1 | 1.00 | Reference |  |
| T2 | 1.49 | 1.20, 1.84 | <0.001 |
| ^1^HR = Hazard Ratio, CI = Confidence Interval | | | |

Supplementary Table 3. Multivariate Cox model adjusted with all covariates for ICU mortality of mimic4_v31 cohort

| **Characteristic** | **HR**^1^ | **95% CI**^1^ | **p-value** |
| --- | --- | --- | --- |
| Group |  |  |  |
| T1 | 1.00 | Reference |  |
| T2 | 1.87 | 1.36, 2.55 | <0.001 |
| Age | 0.99 | 0.98, 1.01 | 0.45 |
| Gender |  |  |  |
| Female | 1.00 | Reference |  |
| Male | 1.07 | 0.78, 1.48 | 0.66 |
| Race |  |  |  |
| Asian | 1.00 | Reference |  |
| Black | 0.97 | 0.38, 2.49 | 0.94 |
| White | 1.10 | 0.46, 2.65 | 0.83 |
| Other | 1.30 | 0.53, 3.16 | 0.56 |
| Weight | 1.00 | 0.99, 1.01 | 0.85 |
| OASIS | 1.06 | 1.03, 1.09 | <0.001 |
| GCS | 0.97 | 0.93, 1.01 | 0.19 |
| Charlson_comorbidity_index | 1.13 | 1.04, 1.22 | 0.002 |
| MV_therapy |  |  |  |
| YES | 1.00 | Reference |  |
| NO | 1.36 | 0.79, 2.32 | 0.26 |
| RRT_therapy |  |  |  |
| YES | 1.00 | Reference |  |
| NO | 1.07 | 0.69, 1.66 | 0.75 |
| Sedative_therapy |  |  |  |
| YES | 1.00 | Reference |  |
| NO | 1.24 | 0.77, 1.99 | 0.37 |
| Vasopressor_therapy |  |  |  |
| YES | 1.00 | Reference |  |
| NO | 0.95 | 0.63, 1.44 | 0.82 |
| COPD |  |  |  |
| YES | 1.00 | Reference |  |
| NO | 1.18 | 0.73, 1.90 | 0.5 |
| CAD |  |  |  |
| YES | 1.00 | Reference |  |
| NO | 1.07 | 0.71, 1.61 | 0.75 |
| HF |  |  |  |
| YES | 1.00 | Reference |  |
| NO | 0.91 | 0.61, 1.36 | 0.65 |
| Hypertension |  |  |  |
| YES | 1.00 | Reference |  |
| NO | 0.79 | 0.56, 1.12 | 0.19 |
| AFIB |  |  |  |
| YES | 1.00 | Reference |  |
| NO | 1.71 | 1.21, 2.42 | 0.002 |
| Stroke |  |  |  |
| YES | 1.00 | Reference |  |
| NO | 1.52 | 1.00, 2.30 | 0.05 |
| T2DM |  |  |  |
| YES | 1.00 | Reference |  |
| NO | 1.78 | 1.21, 2.64 | 0.004 |
| Renal |  |  |  |
| YES | 1.00 | Reference |  |
| NO | 1.15 | 0.73, 1.80 | 0.55 |
| Liver |  |  |  |
| YES | 1.00 | Reference |  |
| NO | 0.88 | 0.59, 1.33 | 0.55 |
| Malignancy |  |  |  |
| YES | 1.00 | Reference |  |
| NO | 3.49 | 0.48, 25.4 | 0.22 |
| Respiratory_rate | 1.00 | 0.98, 1.02 | 0.99 |
| Heart_rate | 1.00 | 0.99, 1.01 | 0.56 |
| MAP | 1.00 | 0.99, 1.01 | 0.8 |
| Temperature | 0.97 | 0.85, 1.11 | 0.68 |
| WBC_count | 1.00 | 1.00, 1.01 | 0.12 |
| Hemoglobin | 0.97 | 0.91, 1.03 | 0.26 |
| Platelet | 1.00 | 1.00, 1.00 | 0.81 |
| pH | 2.08 | 0.47, 9.25 | 0.34 |
| PO2 | 1.00 | 1.00, 1.00 | 0.44 |
| PCO2 | 1.01 | 1.00, 1.02 | 0.04 |
| Lactate | 1.11 | 1.06, 1.16 | <0.001 |
| Creatinine | 0.98 | 0.90, 1.08 | 0.73 |
| ^1^HR = Hazard Ratio, CI = Confidence Interval | | | |

Supplementary Table 4. Multivariate Cox model adjusted with covariates selected by uni-variable analyses for ICU mortality of mimic4_v31 cohort

| **Characteristic** | **HR**^1^ | **95% CI**^1^ | **p-value** |
| --- | --- | --- | --- |
| Group |  |  |  |
| T1 | 1.00 | Reference |  |
| T2 | 1.68 | 1.24, 2.28 | <0.001 |
| OASIS | 1.05 | 1.03, 1.08 | <0.001 |
| GCS | 0.98 | 0.94, 1.02 | 0.26 |
| MV_therapy |  |  |  |
| YES | 1.00 | Reference |  |
| NO | 1.45 | 0.95, 2.21 | 0.08 |
| RRT_therapy |  |  |  |
| YES | 1.00 | Reference |  |
| NO | 1.09 | 0.75, 1.59 | 0.64 |
| Vasopressor_therapy |  |  |  |
| YES | 1.00 | Reference |  |
| NO | 1.09 | 0.80, 1.47 | 0.59 |
| AFIB |  |  |  |
| YES | 1.00 | Reference |  |
| NO | 1.58 | 1.15, 2.17 | 0.005 |
| Liver |  |  |  |
| YES | 1.00 | Reference |  |
| NO | 0.79 | 0.57, 1.09 | 0.15 |
| MAP | 1.00 | 0.99, 1.01 | 0.56 |
| Temperature | 0.94 | 0.83, 1.06 | 0.32 |
| WBC_count | 1.01 | 1.00, 1.01 | 0.04 |
| pH | 0.82 | 0.23, 2.90 | 0.76 |
| Lactate | 1.07 | 1.03, 1.11 | <0.001 |
| ^1^HR = Hazard Ratio, CI = Confidence Interval | | | |

Supplementary Table 5. Multivariate Cox model adjusted with covariates selected by Boruta algorithm for ICU mortality of mimic4_v31 cohort

| **Characteristic** | **HR**^1^ | **95% CI**^1^ | **p-value** |
| --- | --- | --- | --- |
| Group |  |  |  |
| T1 | 1.00 | Reference |  |
| T2 | 1.71 | 1.27, 2.32 | <0.001 |
| Creatinine | 1.00 | 0.92, 1.09 | 0.93 |
| Lactate | 1.07 | 1.03, 1.11 | <0.001 |
| pH | 0.74 | 0.21, 2.58 | 0.64 |
| WBC_count | 1.00 | 1.00, 1.01 | 0.11 |
| Temperature | 0.95 | 0.83, 1.07 | 0.38 |
| MAP | 1.00 | 0.99, 1.01 | 0.6 |
| Respiratory_rate | 1.01 | 0.98, 1.03 | 0.61 |
| RRT_therapy |  |  |  |
| YES | 1.00 | Reference |  |
| NO | 1.12 | 0.73, 1.72 | 0.59 |
| MV_therapy |  |  |  |
| YES | 1.00 | Reference |  |
| NO | 1.37 | 0.90, 2.08 | 0.15 |
| GCS | 0.98 | 0.94, 1.01 | 0.22 |
| OASIS | 1.04 | 1.02, 1.07 | <0.001 |
| ^1^HR = Hazard Ratio, CI = Confidence Interval | | | |

Supplementary Table 6. Multivariate Cox model adjusted with all covariates for In-hospital mortality of mimic4_v31 cohort

| **Characteristic** | **HR**^1^ | **95% CI**^1^ | **p-value** |
| --- | --- | --- | --- |
| Group |  |  |  |
| T1 | 1.00 | Reference |  |
| T2 | 1.33 | 1.05, 1.69 | 0.02 |
| Age | 1.01 | 0.99, 1.02 | 0.39 |
| Gender |  |  |  |
| Female | 1.00 | Reference |  |
| Male | 1.08 | 0.84, 1.38 | 0.56 |
| Race |  |  |  |
| Asian | 1.00 | Reference |  |
| Black | 0.73 | 0.35, 1.54 | 0.41 |
| White | 0.87 | 0.45, 1.71 | 0.7 |
| Other | 1.07 | 0.54, 2.12 | 0.84 |
| Weight | 1.00 | 0.99, 1.01 | 0.92 |
| OASIS | 1.03 | 1.01, 1.05 | 0.008 |
| GCS | 0.98 | 0.95, 1.02 | 0.37 |
| Charlson_comorbidity_index | 1.05 | 0.99, 1.11 | 0.14 |
| MV_therapy |  |  |  |
| YES | 1.00 | Reference |  |
| NO | 0.95 | 0.65, 1.38 | 0.78 |
| RRT_therapy |  |  |  |
| YES | 1.00 | Reference |  |
| NO | 1.11 | 0.76, 1.62 | 0.57 |
| Sedative_therapy |  |  |  |
| YES | 1.00 | Reference |  |
| NO | 1.29 | 0.91, 1.83 | 0.15 |
| Vasopressor_therapy |  |  |  |
| YES | 1.00 | Reference |  |
| NO | 0.92 | 0.68, 1.26 | 0.62 |
| COPD |  |  |  |
| YES | 1.00 | Reference |  |
| NO | 0.81 | 0.58, 1.15 | 0.24 |
| CAD |  |  |  |
| YES | 1.00 | Reference |  |
| NO | 1.06 | 0.78, 1.42 | 0.73 |
| HF |  |  |  |
| YES | 1.00 | Reference |  |
| NO | 1.17 | 0.88, 1.57 | 0.29 |
| Hypertension |  |  |  |
| YES | 1.00 | Reference |  |
| NO | 1.10 | 0.84, 1.43 | 0.5 |
| AFIB |  |  |  |
| YES | 1.00 | Reference |  |
| NO | 1.20 | 0.92, 1.57 | 0.17 |
| Stroke |  |  |  |
| YES | 1.00 | Reference |  |
| NO | 1.15 | 0.83, 1.59 | 0.4 |
| T2DM |  |  |  |
| YES | 1.00 | Reference |  |
| NO | 1.23 | 0.91, 1.65 | 0.17 |
| Renal |  |  |  |
| YES | 1.00 | Reference |  |
| NO | 1.16 | 0.83, 1.62 | 0.4 |
| Liver |  |  |  |
| YES | 1.00 | Reference |  |
| NO | 1.05 | 0.76, 1.44 | 0.79 |
| Malignancy |  |  |  |
| YES | 1.00 | Reference |  |
| NO | 1.99 | 0.79, 5.03 | 0.15 |
| Respiratory_rate | 1.01 | 0.99, 1.03 | 0.36 |
| Heart_rate | 1.00 | 0.99, 1.00 | 0.23 |
| MAP | 0.99 | 0.99, 1.00 | 0.05 |
| Temperature | 0.90 | 0.80, 1.01 | 0.07 |
| WBC_count | 1.01 | 1.00, 1.01 | 0.01 |
| Hemoglobin | 0.95 | 0.91, 1.00 | 0.05 |
| Platelet | 1.00 | 1.00, 1.00 | 0.08 |
| pH | 1.73 | 0.53, 5.71 | 0.36 |
| PO2 | 1.00 | 1.00, 1.00 | 0.57 |
| PCO2 | 1.01 | 1.00, 1.02 | 0.05 |
| Lactate | 1.09 | 1.04, 1.13 | <0.001 |
| Creatinine | 1.04 | 0.97, 1.11 | 0.32 |
| ^1^HR = Hazard Ratio, CI = Confidence Interval | | | |

Supplementary Table 7. Multivariate Cox model adjusted with covariates selected by uni-variable analyses for In-hospital mortality of mimic4_v31 cohort

| **Characteristic** | **HR**^1^ | **95% CI**^1^ | **p-value** |
| --- | --- | --- | --- |
| Group |  |  |  |
| T1 | 1.00 | Reference |  |
| T2 | 1.27 | 1.01, 1.59 | 0.04 |
| OASIS | 1.03 | 1.01, 1.05 | <0.001 |
| GCS | 0.99 | 0.96, 1.02 | 0.56 |
| MV_therapy |  |  |  |
| YES | 1.00 | Reference |  |
| NO | 1.08 | 0.80, 1.47 | 0.6 |
| RRT_therapy |  |  |  |
| YES | 1.00 | Reference |  |
| NO | 1.05 | 0.76, 1.45 | 0.77 |
| Vasopressor_therapy |  |  |  |
| YES | 1.00 | Reference |  |
| NO | 1.03 | 0.81, 1.31 | 0.79 |
| AFIB |  |  |  |
| YES | 1.00 | Reference |  |
| NO | 1.17 | 0.92, 1.49 | 0.2 |
| Liver |  |  |  |
| YES | 1.00 | Reference |  |
| NO | 0.92 | 0.71, 1.18 | 0.52 |
| MAP | 0.99 | 0.99, 1.00 | 0.01 |
| Temperature | 0.88 | 0.80, 0.98 | 0.02 |
| WBC_count | 1.00 | 1.00, 1.01 | 0.06 |
| pH | 0.85 | 0.31, 2.35 | 0.75 |
| Lactate | 1.06 | 1.03, 1.10 | <0.001 |
| ^1^HR = Hazard Ratio, CI = Confidence Interval | | | |

Supplementary Table 8. Multivariate Cox model adjusted with covariates selected by Boruta algorithm for In-hospital mortality of mimic4_v31 cohort

| **Characteristic** | **HR**^1^ | **95% CI**^1^ | **p-value** |
| --- | --- | --- | --- |
| Group |  |  |  |
| T1 | 1.00 | Reference |  |
| T2 | 1.27 | 1.02, 1.59 | 0.04 |
| Creatinine | 1.03 | 0.96, 1.09 | 0.44 |
| Lactate | 1.06 | 1.03, 1.10 | <0.001 |
| pH | 0.85 | 0.31, 2.33 | 0.75 |
| WBC_count | 1.00 | 1.00, 1.01 | 0.1 |
| Temperature | 0.89 | 0.80, 0.98 | 0.02 |
| MAP | 0.99 | 0.99, 1.00 | 0.01 |
| Respiratory_rate | 1.00 | 0.99, 1.02 | 0.58 |
| RRT_therapy |  |  |  |
| YES | 1.00 | Reference |  |
| NO | 1.14 | 0.79, 1.64 | 0.5 |
| MV_therapy |  |  |  |
| YES | 1.00 | Reference |  |
| NO | 1.04 | 0.77, 1.41 | 0.79 |
| GCS | 0.99 | 0.95, 1.02 | 0.42 |
| OASIS | 1.03 | 1.01, 1.04 | 0.004 |
| ^1^HR = Hazard Ratio, CI = Confidence Interval | | | |

Supplementary Table 9. Time Dependent univariable Cox model for ICU mortality of Wenzhou Medical University cohort

| **Characteristic** | **HR**^1^ | **95% CI**^1^ | **p-value** |
| --- | --- | --- | --- |
| Group |  |  |  |
| T1 | 1.00 | Reference |  |
| T2 | 1.89 | 1.47, 2.06 | <0.05 |
| ^1^HR = Hazard Ratio, CI = Confidence Interval | | | |

Supplementary Table 10. Time Dependent univariable Cox model for In-hospital mortality of Wenzhou Medical University cohort

| **Characteristic** | **HR**^1^ | **95% CI**^1^ | **p-value** |
| --- | --- | --- | --- |
| Group |  |  |  |
| T1 | 1.00 | Reference |  |
| T2 | 1.56 | 1.35, 1.98 | <0.01 |
| ^1^HR = Hazard Ratio, CI = Confidence Interval | | | |

**Supplementary Table 11. ICD-10 Code List for Peptic Ulcer Diseases (K25–K28)**

| **ICD-10 Code** | **Description** |
| --- | --- |
| **K25** | **Gastric ulcer** |
| K25.0 | Acute gastric ulcer with hemorrhage |
| K25.1 | Acute gastric ulcer with perforation |
| K25.2 | Acute gastric ulcer with both hemorrhage and perforation |
| K25.3 | Acute gastric ulcer without hemorrhage or perforation |
| K25.4 | Chronic or unspecified gastric ulcer with hemorrhage |
| K25.5 | Chronic or unspecified gastric ulcer with perforation |
| K25.6 | Chronic or unspecified gastric ulcer with both hemorrhage and perforation |
| K25.7 | Chronic gastric ulcer without hemorrhage or perforation |
| K25.9 | Gastric ulcer, unspecified as acute or chronic, without hemorrhage or perforation |
| **K26** | **Duodenal ulcer** |
| K26.0 | Acute duodenal ulcer with hemorrhage |
| K26.1 | Acute duodenal ulcer with perforation |
| K26.2 | Acute duodenal ulcer with both hemorrhage and perforation |
| K26.3 | Acute duodenal ulcer without hemorrhage or perforation |
| K26.4 | Chronic or unspecified duodenal ulcer with hemorrhage |
| K26.5 | Chronic or unspecified duodenal ulcer with perforation |
| K26.6 | Chronic or unspecified duodenal ulcer with both hemorrhage and perforation |
| K26.7 | Chronic duodenal ulcer without hemorrhage or perforation |
| K26.9 | Duodenal ulcer, unspecified as acute or chronic, without hemorrhage or perforation |
| **K27** | **Peptic ulcer, site unspecified** |
| K27.0 | Acute peptic ulcer, site unspecified, with hemorrhage |
| K27.1 | Acute peptic ulcer, site unspecified, with perforation |
| K27.2 | Acute peptic ulcer, site unspecified, with both hemorrhage and perforation |
| K27.3 | Acute peptic ulcer, site unspecified, without hemorrhage or perforation |
| K27.4 | Chronic or unspecified peptic ulcer, site unspecified, with hemorrhage |
| K27.5 | Chronic or unspecified peptic ulcer, site unspecified, with perforation |
| K27.6 | Chronic or unspecified peptic ulcer, site unspecified, with both hemorrhage and perforation |
| K27.7 | Chronic peptic ulcer, site unspecified, without hemorrhage or perforation |
| K27.9 | Peptic ulcer, site unspecified, unspecified as acute or chronic, without hemorrhage or perforation |
| **K28** | **Gastrojejunal ulcer** |
| K28.0 | Acute gastrojejunal ulcer with hemorrhage |
| K28.1 | Acute gastrojejunal ulcer with perforation |
| K28.2 | Acute gastrojejunal ulcer with both hemorrhage and perforation |
| K28.3 | Acute gastrojejunal ulcer without hemorrhage or perforation |
| K28.4 | Chronic or unspecified gastrojejunal ulcer with hemorrhage |
| K28.5 | Chronic or unspecified gastrojejunal ulcer with perforation |
| K28.6 | Chronic or unspecified gastrojejunal ulcer with both hemorrhage and perforation |
| K28.7 | Chronic gastrojejunal ulcer without hemorrhage or perforation |
| K28.9 | Gastrojejunal ulcer, unspecified as acute or chronic, without hemorrhage or perforation |

*Note: Main categories (K25, K26, K27, K28) are shown in bold; subcategories indicate specific clinical presentations.*

**Supplementary Table 12: ICD-9-CM Code List for Peptic Ulcer Diseases (531–534)**

| **ICD-9-CM Code** | **Description (Abbreviated)** |
| --- | --- |
| **531** | **Gastric ulcer** |
| 531.00 | Ac stomach ulcer w hem |
| 531.01 | Ac stomac ulc w hem-obst |
| 531.10 | Ac stomach ulcer w perf |
| 531.11 | Ac stom ulc w perf-obst |
| 531.20 | Ac stomac ulc w hem/perf |
| 531.21 | Ac stom ulc hem/perf-obs |
| 531.30 | Acute stomach ulcer NOS |
| 531.31 | Ac stomach ulc NOS-obstr |
| 531.40 | Chr stomach ulc w hem |
| 531.41 | Chr stom ulc w hem-obstr |
| 531.50 | Chr stomach ulcer w perf |
| 531.51 | Chr stom ulc w perf-obst |
| 531.60 | Chr stomach ulc hem/perf |
| 531.61 | Chr stom ulc hem/perf-ob |
| 531.70 | Chr stomach ulcer NOS |
| 531.71 | Chr stomach ulc NOS-obst |
| 531.90 | Stomach ulcer NOS |
| 531.91 | Stomach ulcer NOS-obstr |
| **532** | **Duodenal ulcer** |
| 532.00 | Ac duodenal ulcer w hem |
| 532.01 | Ac duoden ulc w hem-obst |
| 532.10 | Ac duodenal ulcer w perf |
| 532.11 | Ac duoden ulc w perf-obst |
| 532.20 | Ac duoden ulc w hem/perf |
| 532.21 | Ac duod ulc hem/perf-obs |
| 532.30 | Acute duodenal ulcer NOS |
| 532.31 | Ac duodenal ulc NOS-obst |
| 532.40 | Chr duoden ulcer w hem |
| 532.41 | Chr duoden ulc hem-obstr |
| 532.50 | Chr duoden ulcer w perf |
| 532.51 | Chr duoden ulc perf-obst |
| 532.60 | Chr duoden ulc hem/perf |
| 532.61 | Chr duod ulc hem/perf-ob |
| 532.70 | Chr duodenal ulcer NOS |
| 532.71 | Chr duoden ulc NOS-obstr |
| 532.90 | Duodenal ulcer NOS |
| 532.91 | Duodenal ulcer NOS-obstr |
| **533** | **Peptic ulcer, site unspecified** |
| 533.00 | Ac peptic ulcer w hemorr |
| 533.01 | Ac peptic ulc w hem-obst |
| 533.10 | Ac peptic ulcer w perfor |
| 533.11 | Ac peptic ulc w perf-obs |
| 533.20 | Ac peptic ulc w hem/perf |
| 533.21 | Ac pept ulc hem/perf-obs |
| 533.30 | Acute peptic ulcer NOS |
| 533.31 | Ac peptic ulcer NOS-obst |
| 533.40 | Chr peptic ulcer w hem |
| 533.41 | Chr peptic ulc w hem-obs |
| 533.50 | Chr peptic ulcer w perf |
| 533.51 | Chr peptic ulc perf-obst |
| 533.60 | Chr pept ulc w hem/perf |
| 533.61 | Chr pept ulc hem/perf-ob |
| 533.70 | Chronic peptic ulcer NOS |
| 533.71 | Chr peptic ulcer NOS-obs |
| 533.90 | Peptic ulcer NOS |
| 533.91 | Peptic ulcer NOS-obstruc |
| **534** | **Gastrojejunal ulcer** |
| 534.00 | Ac marginal ulcer w hem |
| 534.01 | Ac margin ulc w hem-obst |
| 534.10 | Ac marginal ulcer w perf |
| 534.11 | Ac margin ulc w perf-obs |
| 534.20 | Ac margin ulc w hem/perf |
| 534.21 | Ac marg ulc hem/perf-obs |
| 534.30 | Ac marginal ulcer NOS |
| 534.31 | Ac marginal ulc NOS-obst |
| 534.40 | Chr marginal ulcer w hem |
| 534.41 | Chr margin ulc w hem-obs |
| 534.50 | Chr marginal ulc w perf |
| 534.51 | Chr margin ulc perf-obst |
| 534.60 | Chr margin ulc hem/perf |
| 534.61 | Chr marg ulc hem/perf-ob |
| 534.70 | Chr marginal ulcer NOS |
| 534.71 | Chr marginal ulc NOS-obs |
| 534.90 | Gastrojejunal ulcer NOS |
| 534.91 | Gastrojejun ulc NOS-obst |

*Note: Main categories (531, 532, 533, 534) are shown in bold. Abbreviations: Ac = Acute; Chr = Chronic; NOS = Not Otherwise Specified; hem = hemorrhage; perf = perforation; obst = obstruction; hem/perf = both hemorrhage and perforation.*

**Supplementary Table S13. Missing Data Proportion for Each Variable in the Study Cohort**

| Variable | Missing (%) |
| --- | --- |
| Demographics |  |
| Age | 0.00 |
| Gender | 0.00 |
| Weight | 1.70 |
| Vital Signs |  |
| Heart rate | 0.10 |
| MAP | 0.10 |
| SBP | 0.10 |
| DBP | 0.10 |
| Temperature | 2.30 |
| Respiratory rate | 0.10 |
| SpO2 | 0.10 |
| Laboratory Parameters |  |
| WBC count | 0.10 |
| RBC count | 0.10 |
| Hemoglobin | 0.10 |
| Platelet count | 0.10 |
| Creatinine | 0.10 |
| BUN | 0.10 |
| Potassium | 0.10 |
| Sodium | 0.10 |
| Chloride | 0.10 |
| Calcium | 0.10 |
| Total bilirubin | 0.10 |
| Direct bilirubin | 0.10 |
| Albumin | 0.10 |
| Total protein | 0.10 |
| Alkaline phosphatase | 0.10 |
| ALT | 0.10 |
| AST | 0.10 |
| PT | 0.10 |
| INR | 0.10 |
| aPTT | 0.10 |
| Fibrinogen | 0.10 |
| D-dimer | 0.10 |
| Lactate | 15.43 |
| pH | 19.84 |
| PaO2 | 42.99 |
| PaCO2 | 46.29 |
| Severity Scores |  |
| GCS | 0.70 |
| SOFA | 0.10 |
| OASIS | 0.10 |
| Treatments |  |
| MV therapy | 0.00 |
| RRT therapy | 0.00 |
| Vasopressor therapy | 0.00 |

**Supplementary Table S14. Comparison of five Cox proportional hazards models for the association of TyG, GV, their interaction, and the composite TyG-GVI index with mortality outcomes.**

*All models adjusted for age, sex, OASIS score, Charlson Comorbidity Index, diabetes, heart failure, renal disease, mechanical ventilation, vasopressor use, lactate, creatinine, and hemoglobin. TyG, triglyceride-glucose index; GV, glycemic variability (coefficient of variation of glucose); TyG-GVI, composite index (TyG × GV); AIC, Akaike Information Criterion; BIC, Bayesian Information Criterion; HR, hazard ratio; CI, confidence interval. Interaction analysis performed in the subsample with available triglyceride data (n = 442). Ridge penalty (λ = 0.05) applied for numerical stability.*

|  | **Model A TyG only** | **Model B GV only** | **Model C Additive** | **Model D Interaction** | **Model E Composite** |
| --- | --- | --- | --- | --- | --- |
| ***ICU Mortality (n = 442, events = 111)*** | | | | | |
| TyG (standardized) | 0.88 (0.74–1.05) | — | 0.88 (0.75–1.05) | 0.93 (0.78–1.13) | — |
| *P value* | 0.166 | — | 0.158 | 0.477 | — |
| GV/CV (standardized) | — | 1.23 (1.04–1.45) | 1.23 (1.04–1.45) | 1.21 (1.02–1.44) | — |
| *P value* | — | 0.016 | 0.016 | 0.031 | — |
| TyG × GV interaction | — | — | — | 0.91 (0.79–1.05) | — |
| *P value* | — | — | — | 0.191 | — |
| TyG-GVI (standardized) | — | — | — | — | 1.18 (1.00–1.40) |
| *P value* | — | — | — | — | 0.056 |

|  | **Model A TyG only** | **Model B GV only** | **Model C Additive** | **Model D Interaction** | **Model E Composite** |
| --- | --- | --- | --- | --- | --- |
| ***In-Hospital Mortality (n = 442, events = 166)*** | | | | | |
| TyG (standardized) | 0.94 (0.80–1.10) | — | 0.93 (0.80–1.08) | 0.96 (0.82–1.13) | — |
| *P value* | 0.432 | — | 0.344 | 0.645 | — |
| GV/CV (standardized) | — | 1.17 (1.02–1.34) | 1.17 (1.02–1.35) | 1.19 (1.03–1.38) | — |
| *P value* | — | 0.029 | 0.024 | 0.018 | — |
| TyG × GV interaction | — | — | — | 0.93 (0.83–1.05) | — |
| *P value* | — | — | — | 0.232 | — |
| TyG-GVI (standardized) | — | — | — | — | 1.14 (0.99–1.30) |
| *P value* | — | — | — | — | 0.062 |

**Supplementary Table S14 (continued).**

Model fit comparison across five Cox proportional hazards models.

|  | **Model A TyG only** | **Model B GV only** | **Model C Additive** | **Model D Interaction** | **Model E Composite** |
| --- | --- | --- | --- | --- | --- |
| ***ICU Mortality*** | | | | | |
| AIC | 1126.60 | 1123.32 | 1123.31 | 1123.57 | 1125.21 |
| BIC | 1179.79 | 1176.50 | 1180.59 | 1184.94 | 1178.40 |
| C-index | 0.7708 | 0.7752 | 0.7757 | 0.7774 | 0.7747 |
| ***In-Hospital Mortality*** | | | | | |
| AIC | 1765.70 | 1762.01 | 1763.11 | 1763.65 | 1763.22 |
| BIC | 1818.88 | 1815.20 | 1820.39 | 1825.02 | 1816.41 |
| C-index | 0.7115 | 0.7171 | 0.7194 | 0.7177 | 0.7162 |

**LRT (Likelihood Ratio Test) for interaction term (Model D vs Model C):**

ICU mortality: χ² = 1.74, df = 1, P = 0.188

In-hospital mortality: χ² = 1.47, df = 1, P = 0.226

**Wald test for interaction term (Model D):**

ICU mortality: Z = −1.31, P = 0.191

In-hospital mortality: Z = −1.19, P = 0.232

**Supplementary Table S15. Incremental predictive performance of adding continuous TyG‑GVI to baseline clinical model**

**Model A: Baseline clinical model (OASIS + age + gender + Charlson Comorbidity Index)**

Model B: Model A + continuous TyG‑GVI

Risk categories defined by predicted mortality probability: low risk < 20%, intermediate risk 20–40%, high risk > 40%

Categorical‑NRI, IDI were computed with 1000 bootstrap resamples to derive 95% CIs and two‑sided p‑values.

Hosmer‑Lemeshow (H‑L) test: p > 0.05 indicates acceptable calibration.

| Metric | ICU mortality | In‑hospital mortality |
| --- | --- | --- |
| Categorical NRI (95% CI) | 0.142 (0.061, 0.224) | 0.108 (0.033, 0.182) |
| P‑value for categorical‑NRI | 0.001 | 0.005 |
| IDI (95% CI) | 0.038 (0.017, 0.063) | 0.027 (0.008, 0.049) |
| P‑value for IDI | <0.001 | 0.006 |
| Hosmer‑Lemeshow P‑value, Model A | 0.216 | 0.134 |
| Hosmer‑Lemeshow P‑value, Model B | 0.347 | 0.228 |

**Supplementary Table S16. Subgroup analysis stratified by pre‑existing diabetes status**

Cox models were adjusted for covariates consistent with Model 2. TyG‑GVI was dichotomized by median (T2 vs T1). P‑interaction: multiplicative interaction term of TyG‑GVI × pre‑existing diabetes status.

| Outcome | Subgroup | n (T1 / T2) | HR (95%CI) for higher TyG‑GVI (T2 vs T1) | P‑value | P‑interaction |
| --- | --- | --- | --- | --- | --- |
| ICU mortality | Without diabetes | 704 (393 / 311) | 2.02 (1.38‑2.96) | <0.001 | 0.227 |
|  | Pre‑existing diabetes | 294 (106 / 188) | 1.44 (0.89‑2.33) | 0.134 |  |
| In‑hospital mortality | Without diabetes | 704 (393 / 311) | 1.48 (1.11‑1.98) | 0.007 | 0.301 |
|  | Pre‑existing diabetes | 294 (106 / 188) | 1.07 (0.74‑1.54) | 0.717 |  |
