# Supplementary material for: Synergistic effect of insulin resistance and glycemic variability on mortality in ICU patients with gastrointestinal bleeding: impact of the TyG-GVI index and development of an explainable web-based clinical calculator
Source: Front Endocrinol (Lausanne). 2026 Sep 4;17:1897817. doi: 10.3389/fendo.2026.1897817 (PMC13585509; doi:10.3389/fendo.2026.1897817)
Supplement: Supplementary file 2 [file SupplementaryFile2.docx]

**Supplementary Appendix S2**

**Complete R Code for TyG-GVI Index Calculation**

*Triglyceride-Glucose Glycemic Variability Index*

This supplementary appendix provides the complete reproducible R code for calculating the TyG-GVI (Triglyceride-Glucose Glycemic Variability Index) as described in the Methods section of the main manuscript. The code includes data extraction queries, unit conversion, outlier handling, coefficient of variation (CV) calculation, and the final composite index derivation.

**Section 1. Key Methodological Notes**

1.1 CV Calculation: Proportion, Not Percentage

The glucose coefficient of variation (CV) was calculated as a proportion (decimal value), not as a percentage:

*CV = Standard Deviation / Mean*

*Example: SD = 17 mg/dL, Mean = 100 mg/dL → CV = 0.17 (not 17%)*

Mathematical justification: The traditional formula CV = (SD/Mean) × 100% is mathematically equivalent to CV = SD/Mean, since 100% = 1. This approach maintains TyG-GVI within a clinically interpretable range (~0.5–3.0).

1.2 Unit Consistency

All glucose values were converted to mg/dL (conversion factor: 1 mmol/L = 18.018 mg/dL). Triglyceride values were also converted to mg/dL (1 mmol/L = 88.57 mg/dL).

1.3 Threshold Verification

The optimal cut-off value of 1.535 was identified through restricted cubic spline (RCS) analysis and corresponds to the 68th percentile of the TyG-GVI distribution. This threshold is consistent with the formula when TyG ≈ 5.50 and CV ≈ 0.28 (representing typical values for high-risk ICU patients with elevated triglycerides and substantial glucose variability).

**Section 2. Complete R Code**

```
# ============================================================================
# TyG-GVI (Triglyceride-Glucose Glycemic Variability Index) Calculation
# Supplementary Appendix S2
# ============================================================================
#
# Study: Synergistic Effect of Insulin Resistance and Glycemic Variability
#        on Mortality in ICU Patients with Gastrointestinal Bleeding
#
# Data source: MIMIC-IV v3.1 database
# Software: R version 4.3.0 or later
# Required packages: dplyr, lubridate, tidyr, survival, survminer
#
# Authors: Yanqiu Jiang, Keyang Guo, Shengshan Xu, Ke-Jie He
# Correspondence: hekejie@stu.xmu.edu.cn
# ============================================================================

# ---- Load Required Packages ----
library(dplyr)
library(lubridate)
library(tidyr)
library(survival)
```

**Step 1. Data Extraction from MIMIC-IV v3.1**

```
# ---- 1.1 Extract glucose measurements within 24h of ICU admission ----
# Glucose item IDs:
#   Lab events (labevents table):
#     50809 - Glucose (mg/dL)
#     50931 - Glucose (mmol/L)
#   Point-of-care / chart events (chartevents table):
#     807   - Glucose finger stick (mg/dL)
#     811   - Glucose (mg/dL)
#     225664 - Glucose (mg/dL)
#     220621 - Glucose (mg/dL)
#     226537 - Glucose (mmol/L)

# Extract all glucose measurements
glucose_all <- DBI::dbGetQuery(con, "
  SELECT
    le.subject_id,
    le.hadm_id,
    ie.icustay_id,
    ie.intime,
    le.charttime,
    le.itemid,
    le.valuenum,
    le.valueuom
  FROM mimiciii_labevents le
  INNER JOIN mimiciii_icustays ie
    ON le.subject_id = ie.subject_id
    AND le.hadm_id = ie.hadm_id
  WHERE le.itemid IN (50809, 50931)
    AND le.charttime >= ie.intime
    AND le.charttime <= ie.intime + INTERVAL '24' HOUR
    AND le.valuenum IS NOT NULL
    AND le.valuenum > 0

  UNION ALL

  SELECT
    ce.subject_id,
    ce.hadm_id,
    ie.icustay_id,
    ie.intime,
    ce.charttime,
    ce.itemid,
    ce.valuenum,
    ce.valueuom
  FROM mimiciii_chartevents ce
  INNER JOIN mimiciii_icustays ie
    ON ce.subject_id = ie.subject_id
    AND ce.icustay_id = ie.icustay_id
  WHERE ce.itemid IN (807, 811, 225664, 220621, 226537)
    AND ce.charttime >= ie.intime
    AND ce.charttime <= ie.intime + INTERVAL '24' HOUR
    AND ce.valuenum IS NOT NULL
    AND ce.valuenum > 0
    AND ce.warning != 1
  ORDER BY subject_id, icustay_id, charttime
")

# ---- 1.2 Extract triglyceride measurements ----
# Triglyceride item IDs:
#   50908 - Triglycerides (mg/dL)
#   51000 - Triglycerides (mmol/L)

# Get first triglyceride measurement (within 24h before or after ICU admission)
tg_data <- DBI::dbGetQuery(con, "
  SELECT DISTINCT ON (le.subject_id, ie.icustay_id)
    le.subject_id,
    ie.icustay_id,
    le.charttime AS tg_time,
    le.itemid AS tg_itemid,
    le.valuenum AS tg_value,
    le.valueuom AS tg_unit
  FROM mimiciii_labevents le
  INNER JOIN mimiciii_icustays ie
    ON le.subject_id = ie.subject_id
    AND le.hadm_id = ie.hadm_id
  WHERE le.itemid IN (50908, 51000)
    AND le.charttime >= ie.intime - INTERVAL '24' HOUR
    AND le.charttime <= ie.intime + INTERVAL '24' HOUR
    AND le.valuenum IS NOT NULL
    AND le.valuenum > 0
  ORDER BY le.subject_id, ie.icustay_id, le.charttime ASC
")
```

**Step 2. Data Cleaning and Unit Conversion**

```
# ---- 2.1 Convert all glucose values to mg/dL ----
# Conversion factor: 1 mmol/L = 18.018 mg/dL

glucose_clean <- glucose_all %>%
  mutate(
    glucose_mg_dl = case_when(
      itemid %in% c(50931, 226537) ~ valuenum * 18.018,
      TRUE ~ valuenum
    )
  )

# ---- 2.2 Outlier handling ----
# Exclude physiologically implausible glucose values:
#   - Lower bound: 20 mg/dL (severe hypoglycemia)
#   - Upper bound: 800 mg/dL (extreme hyperglycemia)

glucose_clean <- glucose_clean %>%
  filter(glucose_mg_dl >= 20 & glucose_mg_dl <= 800)

# ---- 2.3 Convert triglyceride to mg/dL ----
# Conversion factor: 1 mmol/L = 88.57 mg/dL

tg_clean <- tg_data %>%
  mutate(
    tg_mg_dl = case_when(
      tg_itemid == 51000 ~ tg_value * 88.57,
      TRUE ~ tg_value
    )
  )
```

**Step 3. Calculate Glucose Coefficient of Variation (CV)**

```
# ---- 3.1 Calculate per-patient glucose statistics ----
# CV = SD / Mean (as proportion, NOT percentage)
# Requirement: minimum 2 valid glucose measurements per patient

glucose_stats <- glucose_clean %>%
  group_by(subject_id, icustay_id) %>%
  summarise(
    n_glucose = n(),
    glucose_mean = mean(glucose_mg_dl, na.rm = TRUE),
    glucose_sd = sd(glucose_mg_dl, na.rm = TRUE),
    glucose_min = min(glucose_mg_dl, na.rm = TRUE),
    glucose_max = max(glucose_mg_dl, na.rm = TRUE),
    .groups = "drop"
  ) %>%
  filter(n_glucose >= 2) %>%  # Require at least 2 measurements
  mutate(
    # CV as proportion (decimal), NOT percentage
    # Mathematical note: (SD/Mean) × 100% = SD/Mean because 100% = 1
    CV = glucose_sd / glucose_mean
  )

# ---- 3.2 Summary statistics of CV ----
cat("CV (proportion) summary:\n")
summary(glucose_stats$CV)
cat("Mean CV:", mean(glucose_stats$CV, na.rm = TRUE), "\n")
cat("Median CV:", median(glucose_stats$CV, na.rm = TRUE), "\n")
```

**Step 4. Calculate TyG Index**

```
# ---- 4.1 Merge glucose stats with triglyceride data ----

merged_data <- glucose_stats %>%
  inner_join(tg_clean, by = c("subject_id", "icustay_id")) %>%
  # Use the first available glucose measurement as "fasting glucose" proxy
  left_join(
    glucose_clean %>%
      group_by(subject_id, icustay_id) %>%
      slice(1) %>%
      ungroup() %>%
      select(subject_id, icustay_id, first_glucose = glucose_mg_dl),
    by = c("subject_id", "icustay_id")
  )

# ---- 4.2 Calculate TyG ----
# Formula: TyG = ln[fasting triglyceride (mg/dL) × fasting glucose (mg/dL)] / 2
#
# Note: In ICU settings, true fasting status is not applicable.
# We use the first available glucose and triglyceride measurements
# within the study window, consistent with prior MIMIC-IV studies
# in critical care populations.

merged_data <- merged_data %>%
  mutate(
    # TyG index calculation
    TyG = log(tg_mg_dl * first_glucose) / 2
  )

# ---- 4.3 Summary statistics of TyG ----
cat("TyG summary:\n")
summary(merged_data$TyG)
cat("Mean TyG:", mean(merged_data$TyG, na.rm = TRUE), "\n")
cat("Median TyG:", median(merged_data$TyG, na.rm = TRUE), "\n")
```

**Step 5. Calculate TyG-GVI Composite Index**

```
# ---- 5.1 Final calculation ----
# Formula: TyG-GVI = TyG × CV (where CV is proportion, NOT percentage)
#
# Example verification:
#   Patient with TyG = 5.50, CV = 0.36
#   → TyG-GVI = 5.50 × 0.36 = 1.98 (above threshold 1.535)
#   → This corresponds to approximately the 68th percentile

final_data <- merged_data %>%
  filter(!is.na(TyG) & !is.na(CV) & TyG > 0 & CV > 0) %>%
  mutate(
    TyG_GVI = TyG * CV
  )

# ---- 5.2 Validate TyG-GVI distribution ----
cat("\n=== TyG-GVI Distribution ===\n")
cat("N patients:", nrow(final_data), "\n")
cat("Mean TyG-GVI:", mean(final_data$TyG_GVI, na.rm = TRUE), "\n")
cat("Median TyG-GVI:", median(final_data$TyG_GVI, na.rm = TRUE), "\n")
cat("SD TyG-GVI:", sd(final_data$TyG_GVI, na.rm = TRUE), "\n")
cat("Range:", range(final_data$TyG_GVI, na.rm = TRUE), "\n")
cat("IQR:", quantile(final_data$TyG_GVI, c(0.25, 0.75), na.rm = TRUE), "\n")
cat("Threshold 1.535 percentile:",
    mean(final_data$TyG_GVI <= 1.535) * 100, "th percentile\n")
```

**Step 6. Group Assignment Based on Threshold**

```
# Assign patients to high/low TyG-GVI groups based on optimal cut-off
final_data <- final_data %>%
  mutate(
    TyG_GVI_group = case_when(
      TyG_GVI > 1.535 ~ "High TyG-GVI",
      TRUE ~ "Low TyG-GVI"
    )
  )

# Check group distribution
table(final_data$TyG_GVI_group)
prop.table(table(final_data$TyG_GVI_group)) * 100
```

**Step 7. Quality Control and Validation**

```
# ---- 7.1 Verify calculation consistency ----
# Check that TyG-GVI = TyG × CV for all patients

validation <- final_data %>%
  mutate(
    TyG_GVI_check = TyG * CV,
    difference = abs(TyG_GVI - TyG_GVI_check)
  ) %>%
  filter(difference > 1e-10)

if (nrow(validation) == 0) {
  cat("✓ All TyG-GVI calculations verified successfully\n")
} else {
  cat("⚠ Warning:", nrow(validation), "patients with calculation discrepancy\n")
}

# ---- 7.2 Verify threshold classification ----

# Verify that threshold 1.535 is correctly applied
cat("\nThreshold verification:\n")
cat("Patients above 1.535:", sum(final_data$TyG_GVI > 1.535), "\n")
cat("Patients at or below 1.535:", sum(final_data$TyG_GVI <= 1.535), "\n")
cat("Percentage above threshold:",
    round(mean(final_data$TyG_GVI > 1.535) * 100, 1), "%\n")

# ---- 7.3 Example calculations for verification ----
# Provide 3 example patients for manual verification

set.seed(42)
examples <- final_data %>%
  slice_sample(n = 3) %>%
  select(subject_id, icustay_id, n_glucose, glucose_mean,
         glucose_sd, CV, tg_mg_dl, first_glucose, TyG, TyG_GVI)

cat("\n=== Example Calculations for Manual Verification ===\n\n")
for (i in 1:nrow(examples)) {
  ex <- examples[i, ]
  cat(sprintf("Patient %d (ID: %d, ICU stay: %d):\n",
              i, ex$subject_id, ex$icustay_id))
  cat(sprintf("  Glucose measurements: n=%d, mean=%.2f, SD=%.2f mg/dL\n",
              ex$n_glucose, ex$glucose_mean, ex$glucose_sd))
  cat(sprintf("  CV = %.4f / %.4f = %.4f (proportion)\n",
              ex$glucose_sd, ex$glucose_mean, ex$CV))
  cat(sprintf("  Triglyceride = %.2f mg/dL\n", ex$tg_mg_dl))
  cat(sprintf("  First glucose = %.2f mg/dL\n", ex$first_glucose))
  cat(sprintf("  TyG = ln(%.2f × %.2f) / 2 = %.4f\n",
              ex$tg_mg_dl, ex$first_glucose, ex$TyG))
  cat(sprintf("  TyG-GVI = %.4f × %.4f = %.4f\n",
              ex$TyG, ex$CV, ex$TyG_GVI))
  cat(sprintf("  Classification: %s\n\n", ex$TyG_GVI_group))
}
```

**Step 8. Save Results**

```
# Save the final dataset with TyG-GVI
save(final_data, file = "tyg_gvi_final_data.RData")

# Export summary table
write.csv(
  final_data %>%
    select(subject_id, icustay_id, n_glucose, glucose_mean,
           glucose_sd, CV, tg_mg_dl, first_glucose, TyG, TyG_GVI,
           TyG_GVI_group),
  file = "TyG_GVI_calculations.csv",
  row.names = FALSE
)

# ============================================================================
# END OF CODE
# ============================================================================
```

**Section 3. Summary of Key Parameters**

The following table summarizes the key methodological parameters used in the TyG-GVI calculation:

| **Parameter** | **Value / Description** |
| --- | --- |
| Glucose sources | Lab: 50809, 50931; Chart: 807, 811, 225664, 220621, 226537 |
| Triglyceride sources | Lab: 50908 (mg/dL), 51000 (mmol/L) |
| Measurement window | First 24 hours after ICU admission |
| Minimum glucose measurements | ≥ 2 per patient |
| Glucose unit | mg/dL (1 mmol/L = 18.018 mg/dL) |
| Triglyceride unit | mg/dL (1 mmol/L = 88.57 mg/dL) |
| Outlier exclusion (glucose) | < 20 or > 800 mg/dL |
| CV formula | CV = SD / Mean (proportion, NOT percentage) |
| TyG formula | TyG = ln[TG (mg/dL) × glucose (mg/dL)] / 2 |
| TyG-GVI formula | TyG-GVI = TyG × CV |
| Optimal threshold | 1.535 (68th percentile of distribution) |
| Fasting status | Not applicable (ICU setting) |

**Section 4. References**

1. Liu Y, et al. U-shaped association between the glycemic variability and prognosis in hemorrhagic stroke patients: a retrospective cohort study from the MIMIC-IV database. Frontiers in Endocrinology. 2025;16:1546164. doi: 10.3389/fendo.2025.1546164

2. Guerrero-Romero F, et al. The product of triglycerides and glucose (TyG index) for the assessment of insulin resistance: a valid tool in healthy and metabolically abnormal subjects. Endocrine. 2021;73(3):516-523.

3. MIMIC-IV clinical database v3.1. Available at: https://physionet.org/content/mimiciv/3.1/

*This code can be reproduced and modified for research purposes with appropriate attribution to the original study.*
